# BEAST 2: A Software Platform for Bayesian Evolutionary Analysis

**DOI:** 10.1371/journal.pcbi.1003537

**Published:** 2014-04-10

**Authors:** Remco Bouckaert, Joseph Heled, Denise Kühnert, Tim Vaughan, Chieh-Hsi Wu, Dong Xie, Marc A. Suchard, Andrew Rambaut, Alexei J. Drummond

**Affiliations:** 1 Computational Evolution Group, Department of Computer Science, University of Auckland, Auckland, New Zealand; 2 Department of Environmental Systems Science, ETH Zürich, Zürich, Switzerland; 3 Allan Wilson Centre for Molecular Ecology and Evolution, Massey University, Palmerston North, New Zealand; 4 Departments of Biomathematics and Human Genetics, David Geffen School of Medicine, University of California, Los Angeles, Los Angeles, California, United States of America; 5 Department of Biostatistics, School of Public Health, University of California, Los Angeles, Los Angeles, California, United States of America; 6 Institute of Evolutionary Biology, University of Edinburgh, Edinburgh, United Kingdom; 7 Allan Wilson Centre for Molecular Ecology and Evolution, University of Auckland, Auckland, New Zealand; UCSD, United States of America

## Abstract

We present a new open source, extensible and flexible software platform for Bayesian evolutionary analysis called BEAST 2. This software platform is a re-design of the popular BEAST 1 platform to correct structural deficiencies that became evident as the BEAST 1 software evolved. Key among those deficiencies was the lack of post-deployment extensibility. BEAST 2 now has a fully developed package management system that allows third party developers to write additional functionality that can be directly installed to the BEAST 2 analysis platform via a package manager without requiring a new software release of the platform. This package architecture is showcased with a number of recently published new models encompassing birth-death-sampling tree priors, phylodynamics and model averaging for substitution models and site partitioning. A second major improvement is the ability to read/write the entire state of the MCMC chain to/from disk allowing it to be easily shared between multiple instances of the BEAST software. This facilitates checkpointing and better support for multi-processor and high-end computing extensions. Finally, the functionality in new packages can be easily added to the user interface (BEAUti 2) by a simple XML template-based mechanism because BEAST 2 has been re-designed to provide greater integration between the analysis engine and the user interface so that, for example BEAST and BEAUti use exactly the same XML file format.

This is a *PLOS Computational Biology* Software Article.

## Introduction

Bayesian phylogenetics has grown to encompass a number of related inference problems that have at their core the observation of one or more alignments of molecular sequence data from evolutionarily-related samples along with associated information. Depending on the details, the associated information can be in the form of dates/locations of sampling, fossil constraints, or various quantitative or categorical traits associated with the samples for which DNA has been collected.

We use the term *Bayesian evolutionary analysis* to encompass a broad set of analysis types that have at their core a model including one or more phylogenetic or genealogical trees. This includes traditional phylogenetics, but also coalescent-based population genetics, phylodynamics, phylogeography, gene-tree/species-tree reconciliation and related analyses.

The first generation Bayesian Evolutionary Analysis by Sampling Trees (BEAST) package [1], [2] has become a popular platform for solving such problems and takes a modeling philosophy that all of these evolutionary analysis problems share at their core one or more phylogenetic time-trees. A time-tree is a rooted phylogeny in which every node (including the tips) have a time/age associated with them. Typically some times are known (e.g. often the tip times) and some are unknown and must be estimated (e.g. most ancestral divergence times).

This paper describes the overarching design and implementation details of a re-write of the BEAST platform that we have designated BEAST 2, as well as presenting examples of some significant new models developed especially for this new platform.

## Design and Implementation

The design goals for BEAST 2 are to provide a software framework for *Bayesian evolutionary analysis* that is (i) useable, (ii) open and (iii) extensible. Useability implies that the system should be easy to use, well documented, have an intuitive user interface and have a manageable learning curve. By open we mean open access, open source, open input/output formats; all of which facilitate reproducible and verifiable results. Openness also implies that the software should run on many platforms. Extensibility implies a design that is modular and easy to make additions to, especially in a way that does not require re-building and re-deploying the software for each additional feature. All of these design goals are made within a scope that encompasses efficient Bayesian inference and model-based hypothesis testing for sequence data analysis involving phylogenetic time-tree models.

These design goals are broadly similar to those that motivated the development of BEAST 1.x. We embarked on a re-write to address the following shortcomings. The BEAST 1.x design resulted in the emergence of three separately maintained parts: (i) BEAST model/likelihood/operator class hierarchies, (ii) BEAST XML parsing class hierarchy, (iii) BEAUti 1.x class hierarchy and user interface code. Each of these parts requires a substantial learning curve and all must be understood in order to add new functionality. In addition BEAST 1.x has no well developed architecture for third-party extensions without re-building and re-distributing the whole package. Newly developed models have to wait for the next release to become available, unless developers release a derivative of BEAST that would then diverge from the main trunk. Neither of these options is ideal. BEAST 1.x also suffers from patchy documentation and a lack of consistency in the XML input format leading to many idiosyncratic quirks.

### BEAST 2

BEAST 2 provides the same core *Bayesian evolutionary analyses* that have made BEAST 1.x popular. In particular it implements relaxed clocks [3], non-parametric coalescent analysis [4], [5], multispecies coalescent inference [6], phylogeography [7], [8] and others. BEAUti 2 has been designed from the ground up to be seamlessly integrated with the BEAST analysis engine so that all the models developed can easily be added to the user interface without any GUI programming. In addition BEAST 2 has a sequence simulator for simulation studies, post-processing tools (such as LogAnalyzer, LogCombiner, DensiTree [9]) and comprehensive documentation for both users and developers. The advantages of BEAST 2 include (i) the ability to check-point and resume analyses, (ii) reload analysis specification in BEAUti, (iii) package architecture including extensible XML format and template-based GUI development, and (iv) include details of model in trace files.

### BEAST 2 packages

In many ways BEAST 2 can be considered as a library or platform for MCMC-based Bayesian inference and phylogenetics. A BEAST 2 package is a collection of BEASTObject extensions that builds on that platform. Packages make it easier to describe the separate pieces of academic work that have been developed and aid in correct attribution and separation of concerns. The package architecture also streamlines the core class hierarchy in the software making the core software platform easier to learn for new developers. Finally the package architecture facilitates the separation of stable, experimental and dead code. For a detailed tutorial on writing packages we invite readers to read the BEAST 2 website available at http://beast2.org.

### BEAST2 software architecture

**Figure 1:**
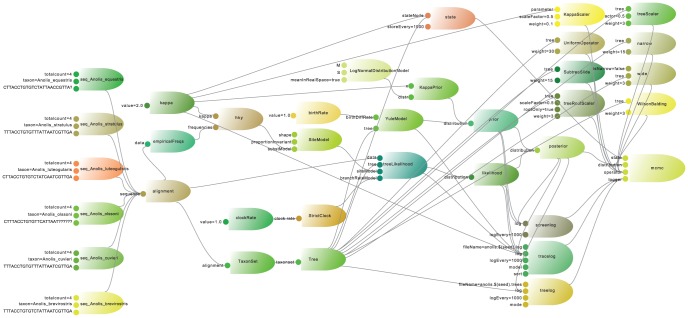
A complete model with six sequences, HKY substitution model, strict clock, Yule tree prior, a number of operators, mainly on the tree, and a few loggers to produce output to a trace file, a tree log and screen output. Note the explicit existence of a State object.

The basic philosophy of BEAST 2 framework is that all user-visible objects are sub-classes of the BEASTObject class. BEASTObject is a top-level class in the core package that provides for (i) inputs, which can be other BEASTObjects or various “primitive” values, (ii) documentation (iii) automatic validation and (iv) XML ‘parsing’. Inputs connect BEASTObjects with other BEASTObjects, allowing a model to be visualized as a directed acyclic graph. Figure 1 shows a model where each rocket represents a BEASTObject and the thrusters the inputs of these BEASTObjects. A lines from a rocket to a thruster indicates the BEASTObject is available as input to the connected BEASTObject. Visualizing the model this way makes it easy for developers to reason about complex models. The BEASTObject class provides for these things both through a set of inherited methods and through a set of conventions on the use of the Input class as instance variables of the BEASTObject class. These conventions support the linking up of BEASTObjects via either XML or the new BEAUti user interface via introspection of a BEASTObject's instance variables. A key role of the BEASTObject class is the automatic conversion of XML input files to a runtime model and analysis specifications in both BEAST and BEAUti 2 without additional XML parsing code.

BEAST 2 is well documented, and the documentation is accessible through the BEAST2 wiki at http://beast2.org/wiki. It contains general information about the programs in the BEAST distribution, frequently asked questions, and a number of tutorials that provide step by step instructions on performing various kinds of analysis, including divergence dating, measurable evolving populations, STAR-BEAST (*BEAST) and phylogeography. Furthermore, there is a mailing list for users and developers and a reference book is available (http://beast2.org/book.html) and is expected to be published in 2014.

## Results

Here we illustrate some of the popular and novel modeling features available on the BEAST2 platform. These features cover the key concerns in an evolutionary analysis: the modeling of the substitution process, and the modeling of the generative and observation processes relating to the time-tree.

BEAST2 implements a range of techniques to improve sampling performance including multi-core parallelization, vector and GPU approaches to fine scale parallelization through the use of the BEAGLE library [10], [11]. BEAST2 can also be made to perform Metropolis-coupled MCMC and the path sampling approach to model comparison [12] is also implemented.

A suite of methods available in former versions of BEAST have been ported to the new framework including ancestral state reconstruction and phylogeography [7], continuous phylogeography [8], and the stochastic Dollo substitution model with various character ascertainment schemes [13].

Two multilocus methods from BEAST1 are available in BEAST2: EBSP [14] reconstructs the effective population size through time, while *BEAST [6] infers the species phylogeny and population size(s).

### New evolutionary models in BEAST 2

One of the significant new additions to BEAST is the implementation of a spike-and-slab [15] mixture model within the BEAST 2 framework [16] that simultaneously estimates the phylogenetic tree, the number of partitions, the assignment of sites to partitions, the nucleotide substitution model and a rate multiplier for each partition. This model facilitates the Bayesian selection over a set of nucleotide substitution models including K80 [17], F81 [18], HKY85 [19], TN93 [20] and GTR [21].

**Figure 2:**
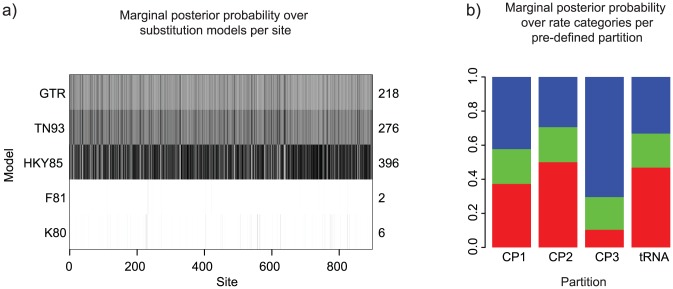
Results from an SDPM2 partition and substitution model averaging analysis of mtDNA from 7 species of primate (data from [22]). (a) The posterior probability distribution of the substitution model given a site. The grid cell at row *i* and column *j* represents the posterior probability of model *i* at site *j*. The darker the shade, the higher the posterior probability. It appears that this data favours models that accommodate a difference in transition and transversion rates and in nucleotide base frequencies. Of those three models, the simplest version (HKY85) is generally preferred. (b) There are four biological partitions in this data set: the three codon positions and a tRNA region. Conditioned on three rate categories (which has the highest posterior probability), the mean posterior proportion of sites in each category for each biological partition is plotted. The categories with faster rates are closer to the top of the bar.

This modelling approach is implemented in the SubstBMA package, and Figure 2 shows the results of applying the SDPM2 model [16] to analyse a small multiple sequence alignment of mtDNA from 12 species of primate [22]. With this model, two independent Dirichlet process priors were applied to the substitution model parameters and site rates. Sites in the same substitution model category are not necessarily in the same rate category. The BEAST2 input file and prior setup of the analysis in Figure 2 is available in the supplementary information Code S2. Figure 2a shows that HKY85 is the model preferred by the most sites, with a posterior expectation of 396 out of 898 sites conforming to this model. Figure 2b shows that as expected, sites in the first and second positions are more likely to be in the lower rate categories than the sites in the third codon position. However it also shows that there is large rate variation within each biological partition.

**Figure 3:**
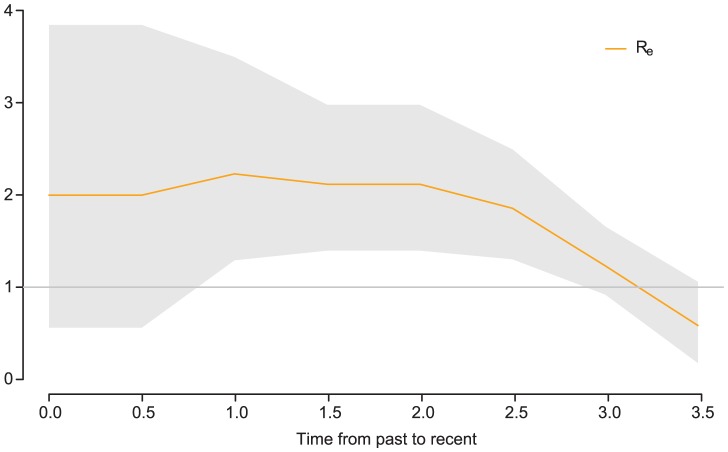
Bayesian estimate of the effective reproduction number *R_e_* over time (from simulated data). Towards the end of the 3.5 years of the underlying epidemic process, *R_e_* decreases below 1, which indicates a declining epidemic.

The BDSKY package contains the birth–death skyline model [23] which employs a piecewise constant birth-death-sampling process to compute the probability density of a phylogeny. In this model, a branching event in the sample tree corresponds to a “birth”, each tip in the tree corresponds to a sampling event, and a death is an unobserved event, i.e. an unsampled recovery or death. Each of these three event types occurs with its own characteristic rate in each interval of the piecewise function. This enables the estimation of epidemiological parameters such as the effective reproduction ratio *R*. In particular, the model permits the reconstruction of changes in *R* over time (Figure 3, see [23] for details). Detecting such changes can help assess the effects of public health measures for the prevention or control of infectious diseases, or to shed light on causes of rapid viral outbreaks. This model can be applied to study the diversification of species. In this scenario a “birth” event in the tree represents a speciation event rather than a transmission event. The Phylodynamics package [24] contains the BDSIR model [25], which allows the reconstruction of viral host population dynamics based on SIR-type models jointly with the phylogenetic inference.

**Figure 4:**
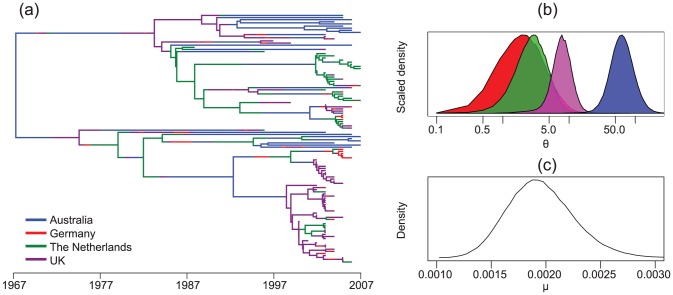
Results from a structured coalescent analysis of a spatially-annotated HCV data set using the MultiTypeTree package. These include (a) a typical tree drawn from the posterior (edge colours represent lineage locations), (b) posterior distributions of *θ* = *N_e_g* for each of the spatially-localized sub-populations and (c) the posterior distribution for the molecular clock rate *μ*_0_.

The MultiTypeTree package [26], [27] implements a structured coalescent model that allows inference of subpopulation sizes and migration rates together with location-annotated genealogies (structured trees) from genetic data. This capability is similar to that of MIGRATE [28]–[30] and is absent from the original BEAST implementation. Additionally, serially sampled data can be used to jointly infer the time-scale of a phylogeny, which is not possible with the current release of MIGRATE. These capabilities are demonstrated in Figure 4, which presents results of using the MultiTypeTree package to analyze a 117 taxon spatially resolved and serially sampled HCV genotype 1a data set [31] under a GTR+*γ*+I substitution model (the BEAST2 input file for this analysis is available in the supplementary information Code S3). This package can be extended to include additional models besides the structured coalescent.

The RBS package contains a reversible-jump based substitution model for nucleotide data [32]. With this substitution model there is no need to choose a substitution model for a given partition, since the RBS model samples the appropriate (mixture of) models given the sequence data. The RBS package also contains an auto-partition model which, as the name suggests, automatically splits an alignment into a fixed number of partitions of contiguous sites. The RBS package is integrated into BEAUti, so its functionality is available in any nucleotide analysis.

The SNAPP package implements a multi-species coalescent for SNP and AFLP data [33]. Unlike *BEAST, which integrates out gene trees through MCMC, SNAPP integrates out the gene trees analytically. SNAPP can be used to determine the species tree and population sizes on the branches in the tree.

The MASTER package uses the BEAST framework to allow users to quickly and easily assemble and simulate a large class of continuous-time discrete-variable stochastic dynamics models [34]. Specific examples include the models commonly found in population genetics, epidemiology, as well as chemistry. The MASTER package allows simulated state space trajectories to be generated, which can in turn be used to estimate the dynamics of moments such as population means and variances. Additionally, the models can be used to generate simulated phylogenetic networks compatible with the population size dynamics. In the case that these networks are tree-like, they can be used to provide starting points for BEAST MCMC calculations.

## Availability and Future Directions

The BEAST 2 platform is an open source project and is anonymously available on a source repository hosted by GitHub at https://github.com/CompEvol/beast2 and supplementary material Code S1. A website providing extra details, documentation, tutorials (supplementary material Text S1, S2, S3) and a draft of an upcoming reference book is also available at http://beast2.org. The platform is open and we expect other researchers to add new methods, models and ideas that will take this framework in directions we cannot predict. Nevertheless, the authors have an interest in developing new models for gene-tree/species-tree reconciliation, species delimitation and species assignment as well as continuing to pursue the development of phylodynamic models that unify statistical phylogenetics and mathematical epidemiology.

## Supporting Information

Code S1 **BEAST 2.1.0 source code as compressed archive.**
(TGZ)

Code S2 **Substitution and partition model averaging example.** Label primate describes the taxonomic scope, SDPM2 is the partition averaging model employed [16] and sc denotes a strict clock analysis. It uses the SubstBMA package, available from http://subst-bma.googlecode.com/.
(XML)

Code S3 **Structured coalescent example.** This example file runs on BEAST 2.1.0 with the MultiTypeTree package (http://compevol.github.io/MultiTypeTree/). The data for the structured coalescent example is made up of Hepatitis C (HCV) NS5B sequences obtained from GenBank as described in [31]. A strict molecular clock is used in conjunction with a GTR+Γ+I substitution model. Log-normal priors (*μ* = 0, *σ* = 1) are applied to each of the population size and migration rate parameters.
(XML)

Text S1 **Tutorial for divergence dating.**
(PDF)

Text S2 **Tutorial for measurable evolving populations.**
(PDF)

Text S3 Tutorial for multi-species coalescent with *BEAST.
(PDF)
