# Supplementary figures and images for: BEAST 2: A Software Platform for Bayesian Evolutionary Analysis
Source: PLoS Comput Biol. 2014 Apr 10;10(4):e1003537. doi: 10.1371/journal.pcbi.1003537 (PMC3985171; doi:10.1371/journal.pcbi.1003537)

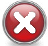

Supplement: Code S1 — BEAST 2.1.0 source code as compressed archive. (TGZ) [file pcbi.1003537.s001.gz › src/beast/app/beauti/cancel.png]

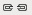

Supplement: Code S1 — BEAST 2.1.0 source code as compressed archive. (TGZ) [file pcbi.1003537.s001.gz › src/beast/app/beauti/unlink.png]

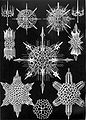

Supplement: Code S1 — BEAST 2.1.0 source code as compressed archive. (TGZ) [file pcbi.1003537.s001.gz › src/beast/app/beauti/1.png]

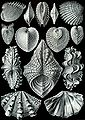

Supplement: Code S1 — BEAST 2.1.0 source code as compressed archive. (TGZ) [file pcbi.1003537.s001.gz › src/beast/app/beauti/7.png]

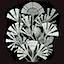

Supplement: Code S1 — BEAST 2.1.0 source code as compressed archive. (TGZ) [file pcbi.1003537.s001.gz › src/beast/app/beauti/0.png]

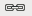

Supplement: Code S1 — BEAST 2.1.0 source code as compressed archive. (TGZ) [file pcbi.1003537.s001.gz › src/beast/app/beauti/link.png]

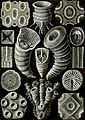

Supplement: Code S1 — BEAST 2.1.0 source code as compressed archive. (TGZ) [file pcbi.1003537.s001.gz › src/beast/app/beauti/3.png]

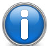

Supplement: Code S1 — BEAST 2.1.0 source code as compressed archive. (TGZ) [file pcbi.1003537.s001.gz › src/beast/app/beauti/finish.png]

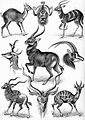

Supplement: Code S1 — BEAST 2.1.0 source code as compressed archive. (TGZ) [file pcbi.1003537.s001.gz › src/beast/app/beauti/6.png]

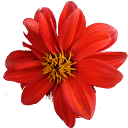

Supplement: Code S1 — BEAST 2.1.0 source code as compressed archive. (TGZ) [file pcbi.1003537.s001.gz › src/beast/app/beauti/10.png]

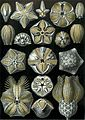

Supplement: Code S1 — BEAST 2.1.0 source code as compressed archive. (TGZ) [file pcbi.1003537.s001.gz › src/beast/app/beauti/5.png]

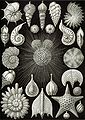

Supplement: Code S1 — BEAST 2.1.0 source code as compressed archive. (TGZ) [file pcbi.1003537.s001.gz › src/beast/app/beauti/2.png]

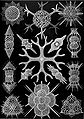

Supplement: Code S1 — BEAST 2.1.0 source code as compressed archive. (TGZ) [file pcbi.1003537.s001.gz › src/beast/app/beauti/8.png]

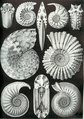

Supplement: Code S1 — BEAST 2.1.0 source code as compressed archive. (TGZ) [file pcbi.1003537.s001.gz › src/beast/app/beauti/4.png]

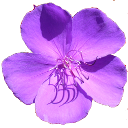

Supplement: Code S1 — BEAST 2.1.0 source code as compressed archive. (TGZ) [file pcbi.1003537.s001.gz › src/beast/app/beauti/11.png]

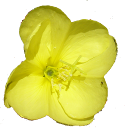

Supplement: Code S1 — BEAST 2.1.0 source code as compressed archive. (TGZ) [file pcbi.1003537.s001.gz › src/beast/app/beauti/12.png]

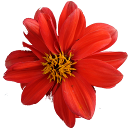

Supplement: Code S1 — BEAST 2.1.0 source code as compressed archive. (TGZ) [file pcbi.1003537.s001.gz › src/beast/app/beauti/13.png]

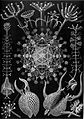

Supplement: Code S1 — BEAST 2.1.0 source code as compressed archive. (TGZ) [file pcbi.1003537.s001.gz › src/beast/app/beauti/9.png]

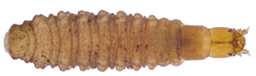

Supplement: Code S1 — BEAST 2.1.0 source code as compressed archive. (TGZ) [file pcbi.1003537.s001.gz › src/beast/app/tools/images/EBSPAnalyser.png]

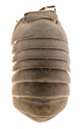

Supplement: Code S1 — BEAST 2.1.0 source code as compressed archive. (TGZ) [file pcbi.1003537.s001.gz › src/beast/app/tools/images/logcombiner.png]

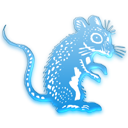

Supplement: Code S1 — BEAST 2.1.0 source code as compressed archive. (TGZ) [file pcbi.1003537.s001.gz › src/beast/app/beastapp/images/beast.png]
