# Supplementary material for: BEAST 2: A Software Platform for Bayesian Evolutionary Analysis
Source: PLoS Comput Biol. 2014 Apr 10;10(4):e1003537. doi: 10.1371/journal.pcbi.1003537 (PMC3985171; doi:10.1371/journal.pcbi.1003537)
Supplement: Text S1 — Tutorial for divergence dating. (PDF) [file pcbi.1003537.s004.pdf]

Divergence Dating Tutorial with BEAST 2.0

Alexei Drummond, Andrew Rambaut and Remco Bouckaert

December 3, 2013

1 Introduction

This tutorial introduces the BEAST software for Bayesian evolutionary analysis through a simple tutorial. The tutorial involves co-estimation of a gene phylogeny and associated divergence times in the presence of calibration information from fossil evidence.

You will need the following software at your disposal:

- **BEAST** - this package contains the BEAST program, BEAUti, TreeAnnotator and other utility programs. This tutorial is written for BEAST v2.0, which has support for multiple partitions. It is available for download from
`http://beast2.cs.auckland.ac.nz/`.
- **Tracer** - this program is used to explore the output of BEAST (and other Bayesian MCMC programs). It graphically and quantitively summarizes the distributions of continuous parameters and provides diagnostic information. At the time of writing, the current version is v1.5. It is available for download from `http://beast.bio.ed.ac.uk/`.
- **FigTree** - this is an application for displaying and printing molecular phylogenies, in particular those obtained using BEAST. At the time of writing, the current version is v1.3.1. It is available for download from `http://tree.bio.ed.ac.uk/`.

This tutorial will guide you through the analysis of an alignment of sequences sampled from twelve primate species (see Figure 1). The goal is to estimate the phylogeny as well as the rate of evolution on each lineage based on divergence times of their host species.

The first step will be to convert a NEXUS file with a DATA or CHARACTERS block into a BEAST XML input file. This is done using the program

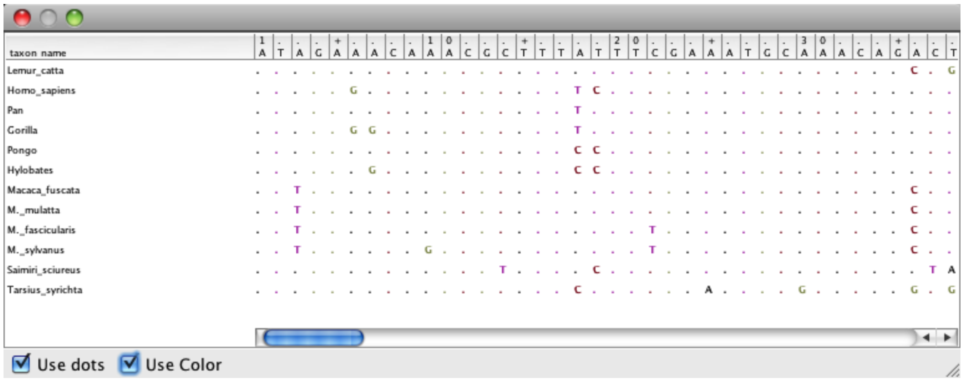

Figure 1: Part of the alignment for primates.

BEAUti (which stands for Bayesian Evolutionary Analysis Utility). This is a user-friendly program for setting the evolutionary model and options for the MCMC analysis. The second step is to actually run BEAST using the input file generated by BEAUTi, which contains the data, model and analysis settings. The final step is to explore the output of BEAST in order to diagnose problems and to summarize the results.

2 BEAUti

The program BEAUti is a user-friendly program for setting the model parameters for BEAST. Run BEAUti by double clicking on its icon. Once running, `BEAUti` will look similar irrespective of which computer system it is running on. For this tutorial, the Mac OS X version is used in the Figures but the Linux and Windows versions will have the same layout and functionality.

2.1 Loading the NEXUS file

To load a NEXUS format alignment, simply select the `Import Alignment...` option from the File menu.

The example file called `primates-mtDNA.nex` is in the `examples/nexus/` directory of the directory where BEAST was installed. This file contains an alignment of sequences of 12 species of primates.

Once loaded, five character partitions are displayed in the main panel (Figure 2). **You must remove the 'coding' partition before continuing to the next step as it refers to the same nucleotides as**

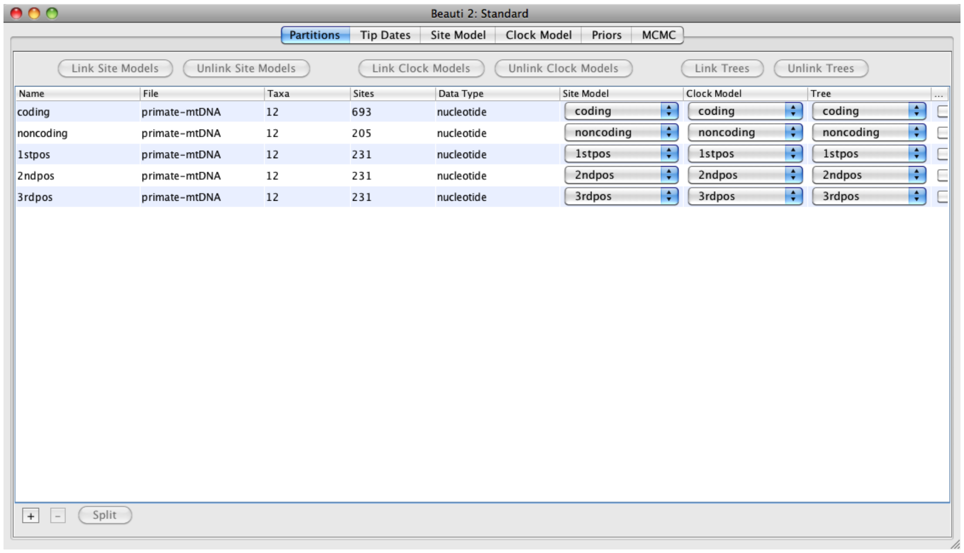

Figure 2: A screenshot of the data tab in BEAUti.

partitions '1stpos', '2ndpos' and '3rdpos'. To remove the 'coding' partition select the row and click the '-' button at the bottom of the table.

Link/Unlink partition models

At this point we will need to link the clock model and tree. In the **Partitions** panel, select all four partitions in the table (or none, by default all partitions are affected) and click the `Link Tree Models` button and then the `Link Clock Models` button (see Figure 3). Then click on the first drop-down menu in the Clock Model column and rename the shared clock model to 'clock'. Likewise rename the shared tree to 'tree'. This will make following options and generated log files more easy to read.

2.2 Setting the substitution model

The next step is to set up the substitution model. First we will temporarily link the site models in the Partitions panel so that we can change the model of all partitions simultaneously. Then, select the **Site Models** tab at the top of the main window. This will reveal the evolutionary model settings for BEAST. The options available depend on whether the data are nucleotides, or amino acids, binary data, or general data. The settings that will appear

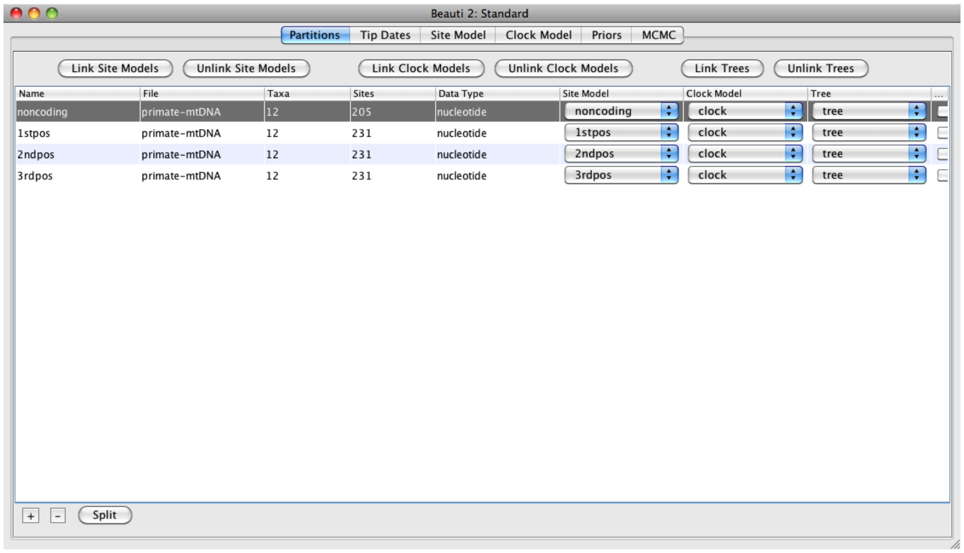

Figure 3: A screenshot of the Partitions tab in BEAUti after linking and renaming the clock model and tree.

after loading the primate nucleotide alignment will be the default values for nucleotide data so we need to make some changes.

Most of the models should be familiar to you. First, set the **Gamma Category Count** to 4 and then check the 'estimate' box for the **Shape** parameter. This will allow rate variation between sites in each partition to be modeled. Then select **HKY** from the **Subst Model** drop-down menu (Figure 4) and select **Empirical** from the **Frequencies** drop-down menu. This will fix the frequencies to the proportions observed in the data (for each partition individually, once we unlink the site models again). This approach means that we can get a good fit to the data without explicitly estimating these parameters. We do it here simply to make the log files a bit shorter and more readable in later parts of the exercise. Finally check the 'estimate' box for the **Substitution rate** parameter and select the **Fix mean mutation rate** check box. This will allow the individual partitions to have their relative rates estimated once we unlink the site models.

Now, return to the 'Partitions' panel and unlink the site models so that each partition has its own named site model with independent substitution model parameters and relative rate.

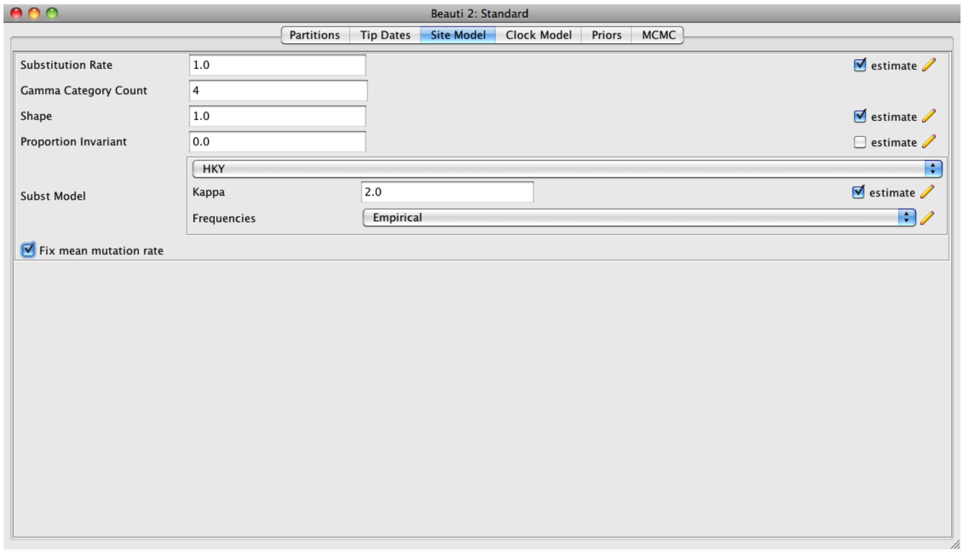

Figure 4: A screenshot of the site model tab in BEAUti.

2.3 Setting the clock model

The next step is to select the **Clock Models** tab at the top of the main window. This is where we select the molecular clock model. For this exercise we are going to leave the selection at the *default* value of a Strict molecular clock, because this data is very clock-like and does not need rate variation among branches to be included in the model.

2.4 Priors

The **Priors** tab allows priors to be specified for each parameter in the model. The model selections made in the site model and clock model tabs, result in the inclusion of various parameters in the model, and these are shown in the priors tab (see Figure 5).

Here we also specify that we wish to use the Calibrated Yule model [1] as the tree prior. This is a simple model of speciation that is generally more appropriate when considering sequences from different species. Select this from the **Tree prior** dropdown menu.

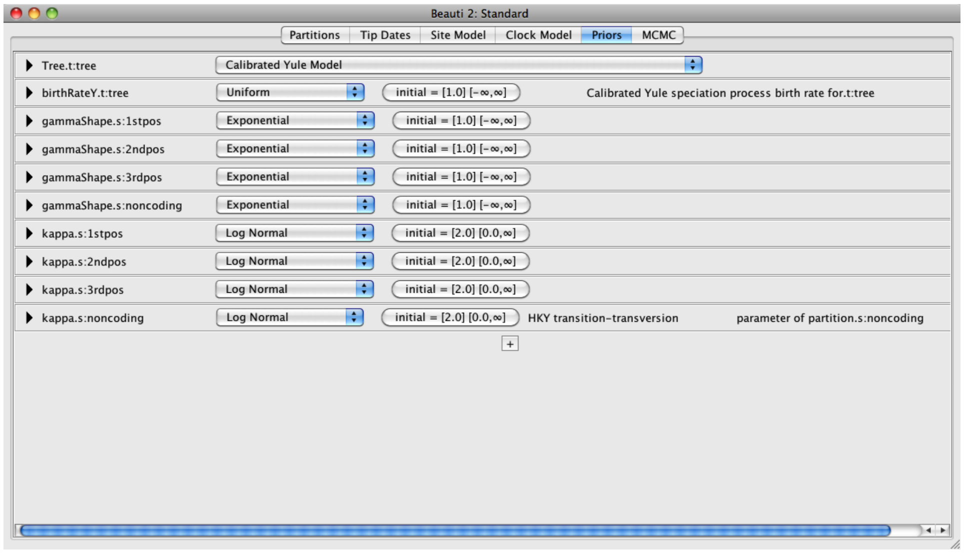

Figure 5: A screenshot of the Priors tab in BEAUti.

2.4.1 Defining the calibration node

To define an extra prior, press the small + button below list of priors. You will see a dialog that allows you to define a subset of the taxa in the phylogenetic tree. Once you have created a taxa set you will be able to add calibration information for its most recent common ancestor (MRCA) later on.

Name the taxa set by filling in the taxon set label entry. Call it `human-chimp` (it will contain the taxa for *Homo sapiens* and *Pan*). In next list below you will see the available taxa. Select each of the two taxa in turn and press the >> arrow button. Click OK and the newly defined taxa set will be added in to the prior list. As this is a calibrated node to be used in conjunction with the Calibrated Yule prior, monophyly must be enforced, so select the checkbox marked `Monophyletic`. This will constrain the tree topology so that the human-chimp grouping is kept monophyletic during the course of the MCMC analysis.

We now need to specify a prior distribution on the calibrated node, based on our prior fossil knowledge. This is known as calibrating our tree. Select the **Normal** distribution from the drop down menu to the right of the newly added `human-chimp.prior`. Click on the black triangle to the right and a graph of the probability density function will appear, along with parameters for the normal distribution. We are going to specify a normal distribution

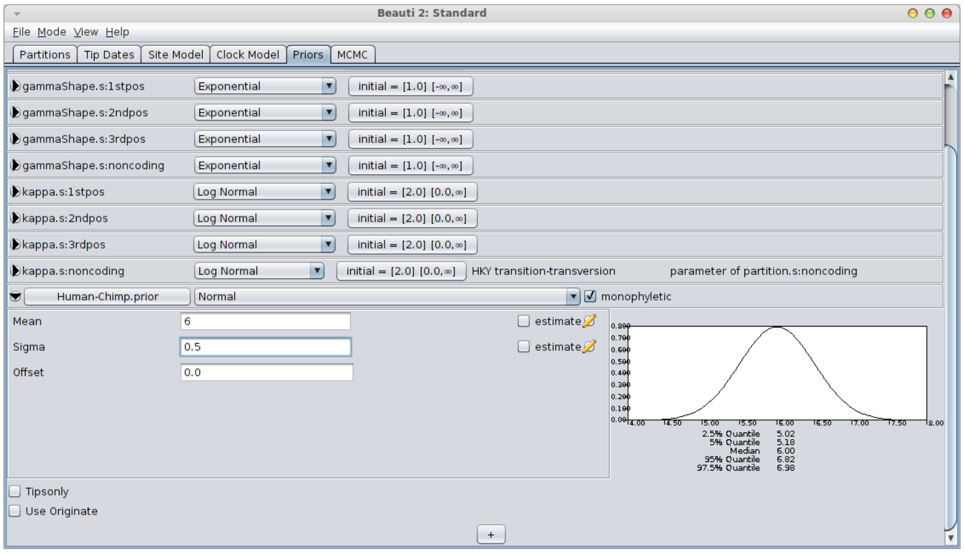

Figure 6: A screenshot of the calibration prior options in the Priors panel in BEAUti.

centered at 6 million years with a standard deviation of 0.5 million years. This will give a central 95% range of about 5-7 My. This roughly corresponds to the current consensus estimate of the date of the most recent common ancestor of humans and chimpanzees (Figure 6).

Finally we will also specify some diffuse "uninformative" but proper priors on the overall molecular clock rate (`clockRate`) and the speciation rate (`birthRateY`) of the Yule tree prior. For each of these parameters select **Gamma** from the drop-down menu and using the arrow button to the right, expand the view to reveal the parameters of the Gamma prior. For both the clock rate and the Yule birth rate set the Alpha (shape) parameter to 0.001 and the Beta (scale) parameter to 1000.

2.5 Setting the MCMC options

The next tab, **MCMC**, provides more general settings to control the length of the MCMC run and the file names.

Firstly we have the **Chain Length**. This is the number of steps the MCMC will make in the chain before finishing. How long this should be depends on the size of the data set, the complexity of the model and the quality of answer required. The default value of 10,000,000 is entirely arbitrary and should be adjusted according to the size of your data set. For

this data set let's initially set the chain length to 1,000,000 as this will run reasonably quickly on most modern computers (a few minutes).

We will leave the **Store Every** and **Pre Burnin** fields set to their default values. Below these are the details of the log files. Each one can be expanded by clicking the arrow to the right

The next options specify how often the parameter values in the Markov chain should be displayed on the screen and recorded in the log file. The screen output is simply for monitoring the programs progress so can be set to any value (although if set too small, the sheer quantity of information being displayed on the screen will actually slow the program down). For the log file, the value should be set relative to the total length of the chain. Sampling too often will result in very large files with little extra benefit in terms of the accuracy of the analysis. Sample too infrequently and the log file will not record sufficient information about the distributions of the parameters. You probably want to aim to store no more than 10,000 samples so this should be set to no less than chain length / 10000.

For this exercise we will set the screen log to 1000 and the trace log to 200. The final two options give the file names of the log files for the sampled parameters and the trees. These will be set to a default based on the name of the imported NEXUS file.

- If you are using the Windows operating system then we suggest you add the suffix `.txt` to both of these (so, `Primates.log.txt` and `Primates.trees.txt`) so that Windows recognizes these as text files.

2.6 Generating the BEAST XML file

We are now ready to create the BEAST XML file. To do this, select the **Save** option from the **File** menu. Check the default priors, and save the file with an appropriate name (we usually end the filename with `.xml`, i.e., `Primates.xml`). We are now ready to run the file through BEAST.

3 Running BEAST

Now run BEAST and when it asks for an input file, provide your newly created XML file as input. BEAST will then run until it has finished reporting information to the screen. The actual results files are save to the disk in the same location as your input file. The output to the screen will look something like this:

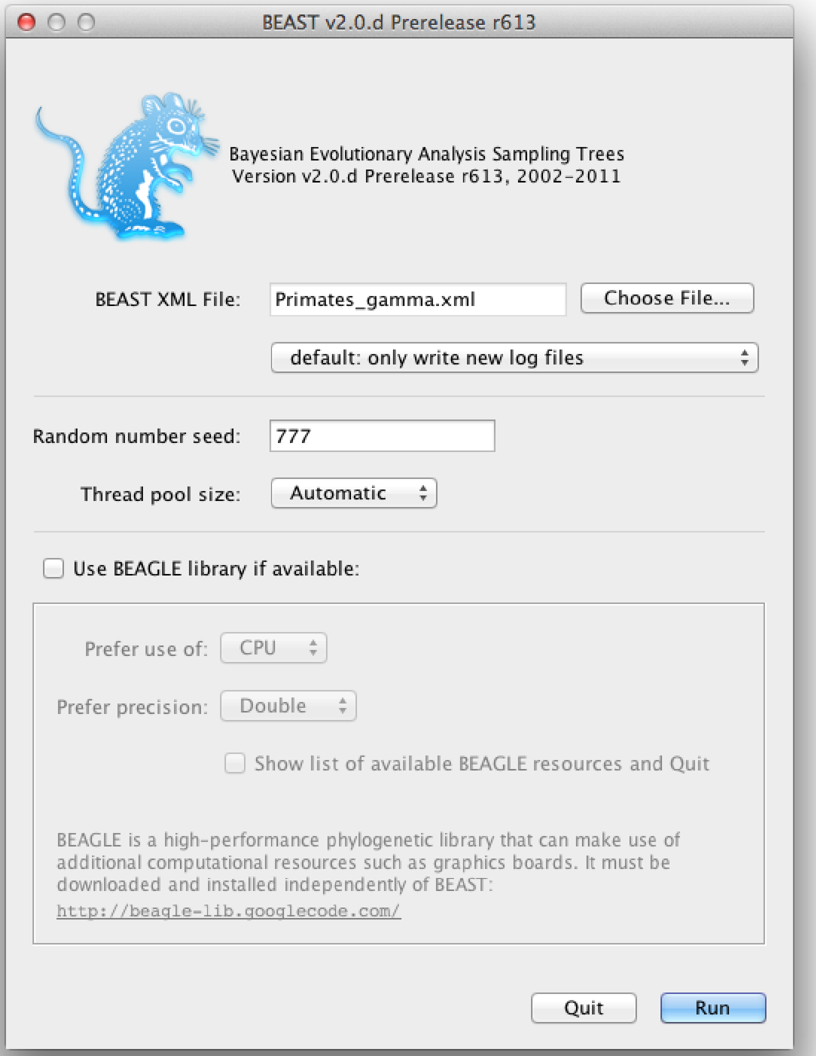

Figure 7: A screenshot of BEAST.

```
          BEAST v2.0.d Prerelease r613, 2002-2011
       Bayesian Evolutionary Analysis Sampling Trees
                 Designed and developed by
Remco Bouckaert, Alexei J. Drummond, Andrew Rambaut and Marc A. Suchard

               Department of Computer Science
                   University of Auckland
                  remco@cs.auckland.ac.nz
                  alexei@cs.auckland.ac.nz

             Institute of Evolutionary Biology
                  University of Edinburgh
                     a.rambaut@ed.ac.uk

              David Geffen School of Medicine
           University of California, Los Angeles
                     msuchard@ucla.edu

                Downloads, Help & Resources:
               http://beast2.cs.auckland.ac.nz

Source code distributed under the GNU Lesser General Public License:
               http://code.google.com/p/beast2

                     BEAST developers:
Alex Alekseyenko, Trevor Bedford, Erik Bloomquist, Joseph Heled,
Sebastian Hoehna, Denise Kuehnert, Philippe Lemey, Wai Lok Sibon Li,
Gerton Lunter, Sidney Markowitz, Vladimir Minin, Michael Defoin Platel,
           Oliver Pybus, Chieh-Hsi Wu, Walter Xie

                         Thanks to:
     Roald Forsberg, Beth Shapiro and Korbinian Strimmer


Random number seed: 777

12 taxa
898 sites
413 patterns
TreeLikelihood uses beast.evolution.likelihood.BeerLikelihoodCore4
TreeLikelihood uses beast.evolution.likelihood.BeerLikelihoodCore4
TreeLikelihood uses beast.evolution.likelihood.BeerLikelihoodCore4
TreeLikelihood uses beast.evolution.likelihood.BeerLikelihoodCore4
=======================================================
Please cite the following when publishing this model:

A prototype for BEAST 2.0: The computational science of evolutionary software. Bouckaert, Drummond, Rambaut, Alekse

Heled J, Drummond AJ. Calibrated Tree Priors for Relaxed Phylogenetics and Divergence Time Estimation. Syst Biol (2

Hasegawa, M., Kishino, H and Yano, T. 1985. Dating the human-ape splitting by a molecular clock of mitochondrial DN


=======================================================
Trying to write file primate-mtDNA.777.log but the file already exists (perhaps use the -overwrite flag?).
Overwrite (Y/N)?:
```

```
Y
Writing file primate-mtDNA.777.log
         Sample      posterior ESS(posterior)     likelihood          prior
Writing file primate-mtDNA.tree.777.trees
              0     -7766.9711              N     -7688.4922       -78.4789 --
          10000     -5527.1265        2.0       -5453.0299       -74.0966 --
          20000     -5521.2666        3.0       -5446.4954       -74.7711 --
          30000     -5518.7901        4.0       -5442.6380       -76.1520 --
          40000     -5514.6676        5.0       -5438.3693       -76.2982 --
          50000     -5522.7987        6.0       -5447.3333       -75.4654 --
          60000     -5513.6936        7.0       -5440.6748       -73.0187 2m50s/Msamples
          ...
        9990000     -5512.1732      739.1       -5441.1958       -70.9773 2m49s/Msamples
       10000000     -5515.2321      734.5       -5437.9182       -77.3138 2m49s/Msamples
Operator                                                              Tuning #accept #reject
#total acceptance rate
ScaleOperator_treeScaler.t:tree                                        0.728  75940 281958
357898 0.212
ScaleOperator_treeRootScaler.t:tree                                    0.581  48659 309158
357817 0.136
Uniform_UniformOperator.t:tree                                                799104 2781229
3580333 0.223
SubtreeSlide_SubtreeSlide.t:tree                                       10.01  450154 1339576
1789730 0.252
Exchange_narrow.t:tree                                                        1368 1787165
1788533 0.001
Exchange_wide.t:tree                                                          25 357913
357938 0
WilsonBalding_WilsonBalding.t:tree                                            14 358742
358756 0
ScaleOperator_gammaShapeScaler.s:noncoding                             0.369  2843 8998
11841 0.24
ScaleOperator_KappaScaler.s:noncoding                                  0.352  2950 8870
11820 0.25
DeltaExchangeOperator_FixMeanMutationRatesOperator                     0.340  35796 203561
239357 0.15
ScaleOperator_KappaScaler.s:1stpos                                     0.420  2713 9297
12010 0.226
ScaleOperator_gammaShapeScaler.s:1stpos                                0.419  3266 8762
12028 0.272
ScaleOperator_KappaScaler.s:2ndpos                                     0.324  2886 8933
11819 0.244
ScaleOperator_gammaShapeScaler.s:2ndpos                                0.278  2984 9046
12030 0.248
ScaleOperator_KappaScaler.s:3rdpos                                     0.541  2622 9246
11868 0.221
ScaleOperator_gammaShapeScaler.s:3rdpos                                0.308  3343 8577
11920 0.28
ScaleOperator_CalibratedYuleBirthRateScaler.t:tree                     0.249  98194 258404
356598 0.275
ScaleOperator_StrictClockRateScaler.c:clock                            0.704  82888 276401
359289 0.231
UpDownOperator_strictClockUpDownOperator.c:clock                       0.600  85379 273037
358416 0.238
Total calculation time: 1710.509 seconds
```

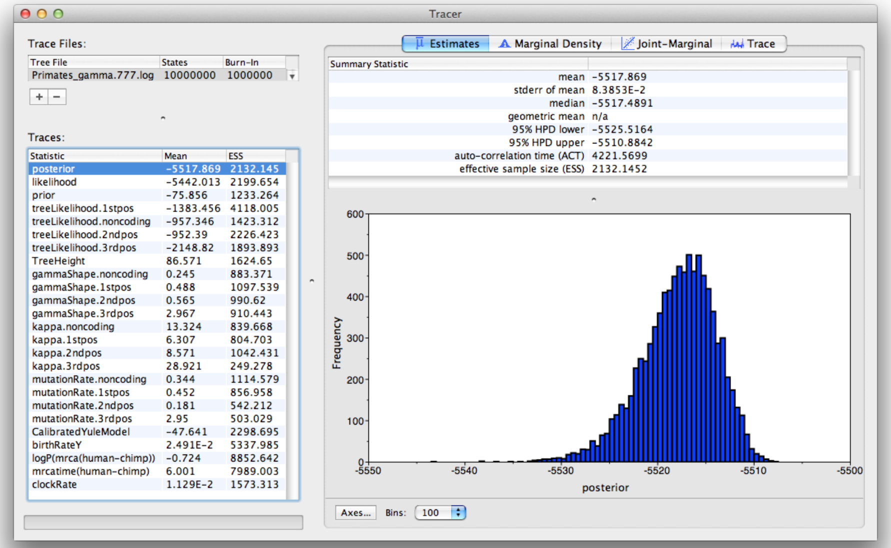

Figure 8: A screenshot of Tracer v1.5.

4 Analyzing the results

Run the program called **Tracer** to analyze the output of BEAST. When the main window has opened, choose **Import Trace File...** from the **File** menu and select the file that BEAST has created called `Primates.log` (Figure 8).

Remember that MCMC is a stochastic algorithm so the actual numbers will not be exactly the same as those depicted in the figure.

On the left hand side is a list of the different quantities that BEAST has logged to file. There are traces for the posterior (this is the natural logarithm of the product of the tree likelihood and the prior density), and the continuous parameters. Selecting a trace on the left brings up analyses for this trace on the right hand side depending on tab that is selected. When first opened, the 'posterior' trace is selected and various statistics of this trace are shown under the Estimates tab. In the top right of the window is a table of calculated statistics for the selected trace.

Select the `clockRate` parameter in the lefthand list to look at the average rate of evolution (averaged over the whole tree and all sites). Tracer will plot a (marginal posterior) histogram for the selected statistic and also give you summary statistics such as the mean and median. The 95% HPD stands for

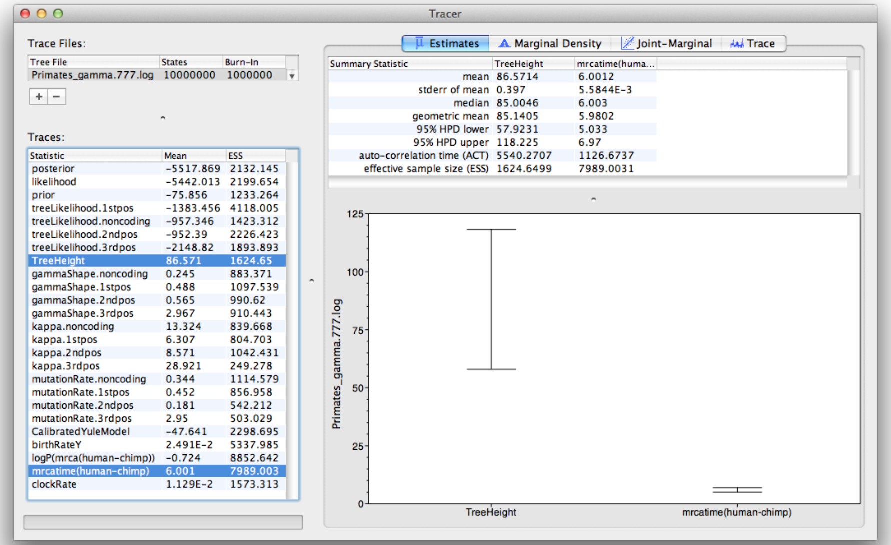

Figure 9: A screenshot of the 95% HPD intervals of the root height and the user-specified (human-chimp) MRCA in Tracer.

highest posterior density interval and represents the most compact interval on the selected parameter that contains 95% of the posterior probability. It can be loosely thought of as a Bayesian analog to a confidence interval. The `TreeHeight` parameter gives the marginal posterior distribution of the age of the root of the entire tree.

Select the `TreeHeight` parameter and then Ctrl-click `mrcatime(human-chimp)` (Command-click on Mac OS X). This will show a display of the age of the root and the calibration MRCA we specified earlier in BEAUti. You can verify that the divergence that we used to calibrate the tree (`mrcatime(human-chimp)`) has a posterior distribution that matches the prior distribution we specified (Figure 9).

Questions

What is the estimated rate of molecular evolution for this gene tree (include the 95% HPD interval)?

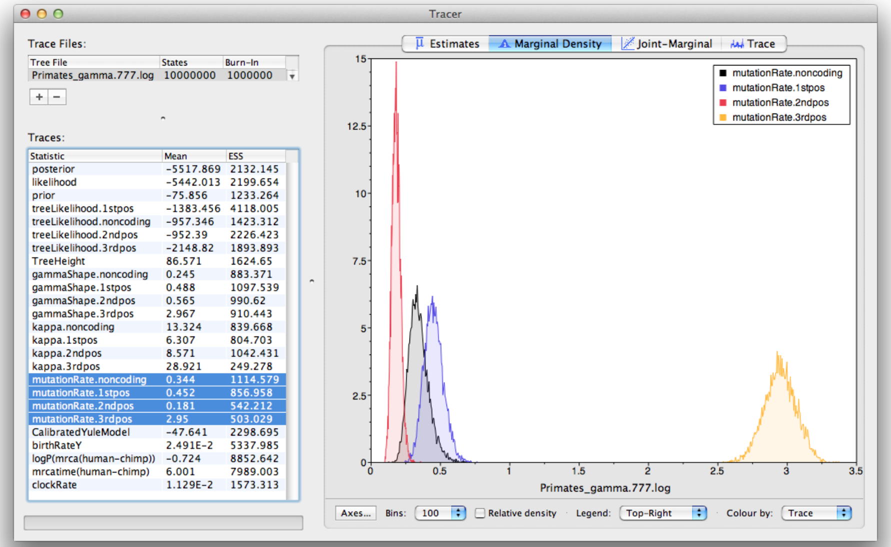

Figure 10: A screenshot of the marginal posterior densities of the relative substitution rates of the four partitions (relative to the site-weighted mean rate). This plot shows that codon positions 1 and 2 have substantially different rates (0.452 versus 0.181) and both are far slower than codon position 3 with a relative rate of 2.95. The noncoding partition has a rate intermediate between codon positions 1 and 2 (0.344). Taken together this result suggests strong purifying selection in both the coding and noncoding regions of the alignment.

What sources of error does this estimate include?

How old is the root of the tree (give the mean and the 95% HPD range)?

5 Obtaining an estimate of the phylogenetic tree

BEAST also produces a posterior sample of phylogenetic time-trees along with its sample of parameter estimates. These need to be summarized using the program **TreeAnnotator**. This will take the set of trees and find the best supported one. It will then annotate this representative summary tree with the mean ages of all the nodes and the corresponding 95% HPD ranges. It will also calculate the posterior clade probability for each node. Run the TreeAnnotator program and set it up as depicted in Figure 11.

The burnin is the number of trees to remove from the start of the sample. Unlike **Tracer** which specifies the number of steps as a burnin, in **TreeAnnotator** you need to specify the actual number of trees. For this run, you specified a chain length of 1,000,000 steps sampling every 200 steps. Thus the trees file will contain 5000 trees and so to specify a 1% burnin use the value 50.

The **Posterior probability limit** option specifies a limit such that if a node is found at less than this frequency in the sample of trees (i.e., has a posterior probability less than this limit), it will not be annotated. The default of 0.5 means that only nodes seen in the majority of trees will be annotated. Set this to zero to annotate all nodes.

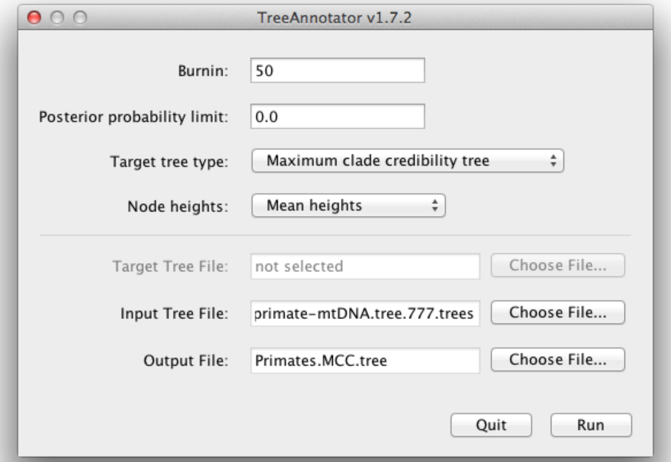

Figure 11: A screenshot of TreeAnnotator.

For **Target tree type** you can either choose a specific tree from a file or ask TreeAnnotator to find a tree in your sample. The default option, **Maximum clade credibility tree**, finds the tree with the highest product of the posterior probability of all its nodes.

Choose **Mean heights** for node heights. This sets the heights (ages) of each node in the tree to the mean height across the entire sample of trees for that clade.

For the input file, select the trees file that BEAST created and select a file for the output (here we called it `Primates.MCC.tree`).

Now press Run and wait for the program to finish.

6 Visualizing the tree estimate

Finally, we can visualize the tree in another program called **FigTree**. Run this program, and open the `Primates.MCC.tree` file by using the Open command in the File menu. The tree should appear. You can now try selecting some of the options in the control panel on the left. Try selecting **Node Bars** to get node age error bars. Also turn on **Branch Labels** and select **posterior** to get it to display the posterior probability for each node. If you use a non strict clock model then under **Appearance** you can also

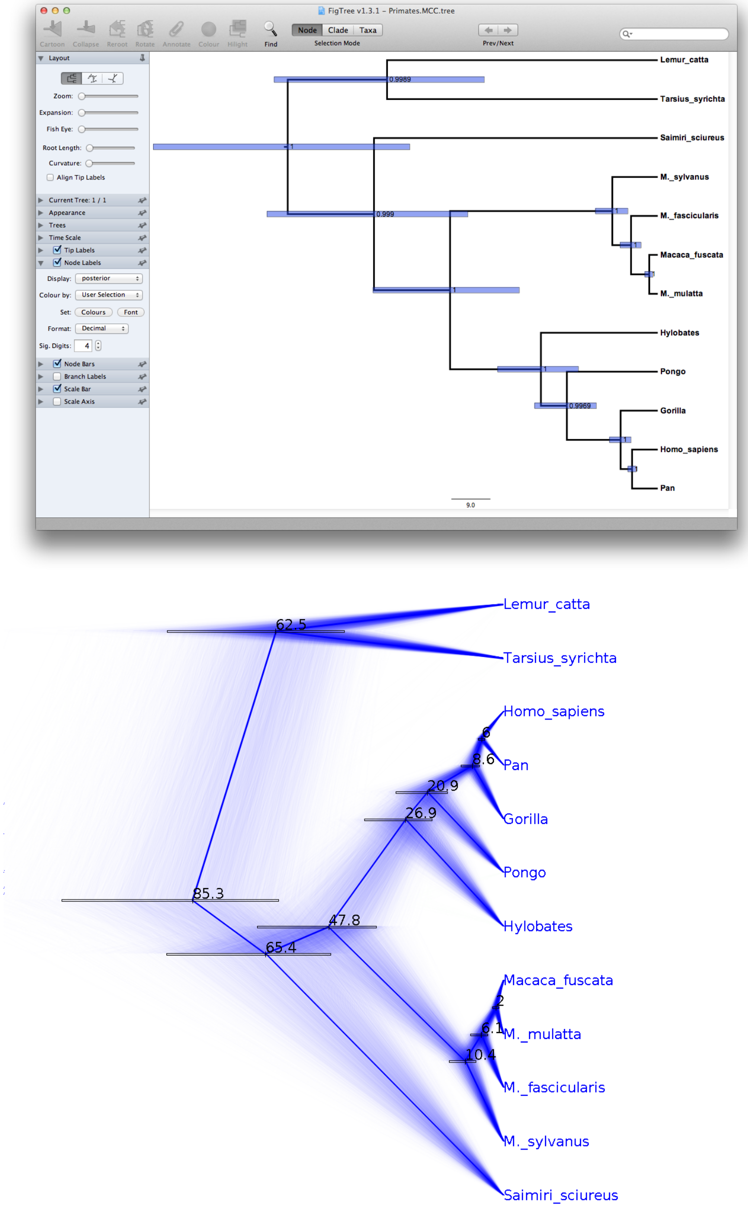

Figure 12: A screenshot of FigTree and DensiTree.

tell FigTree to colour the branches by the rate. You should end up with something similar to Figure 12.

An alternative view of the tree can be made with DensiTree, which is part of Beast 2. The advantage of DensiTree is that it is able to visualize both uncertainty in node heights and uncertainty in topology. For this particular dataset, the dominant topology is present in more than 99% of the samples. So, we conclude that this analysis results in a very high consensus on topology (Figure 12).

Questions

Does the rate of evolution differ substantially amongst different lineages in the tree?

DensiTree has a clade bar (Menu Window/View clade toolbar) to show information on clades.

What is the support for the clade [Homo_sapiens, Pan, Gorilla, Hylobates]?

You can browse through the topologies in DensiTree using the Browse menu. The most popular topology has a support of over 99%.

What is the support for the second most popular topology?

Under the help menu, DensiTree shows some information.
How many topologies are in the tree set?

7 Comparing your results to the prior

Using BEAUti, set up the same analysis but under the MCMC options, select the **Sample from prior only** option. This will allow you to visualize the full prior distribution in the absence of your sequence data. Summarize the trees from the full prior distribution and compare the summary to the posterior summary tree.

References

[1] Joseph Heled and Alexei J Drummond, *Calibrated tree priors for relaxed phylogenetics and divergence time estimation*, Syst Biol **61** (2012), no. 1, 138–49.
