# Supplementary material for: BEAST 2: A Software Platform for Bayesian Evolutionary Analysis
Source: PLoS Comput Biol. 2014 Apr 10;10(4):e1003537. doi: 10.1371/journal.pcbi.1003537 (PMC3985171; doi:10.1371/journal.pcbi.1003537)
Supplement: Text S2 — Tutorial for measurable evolving populations. (PDF) [file pcbi.1003537.s005.pdf]

# Measurably evolving populations

Alexei J Drummond and Remco Bouckaert

December 3, 2013

## 1 Time-stamped data

This tutorial estimates the rate of evolution from a set of virus sequences which have been isolated at different points in time (heterochronous or time-stamped data). The data are 129 sequences from the G (attachment protein) gene of human respiratory syncytial virus subgroup A (RSVA) from various parts of the world with isolation dates ranging from 1956-2002 [2, 1]. RSVA causes infections of the lower respiratory tract causing symptoms that are often indistinguishable from the common cold. By age 3, nearly all children will be infected and a small percentage ($< 3\%$) will develop more serious inflammation of the bronchioles requiring hospitalisation.

The aim of this tutorial is to obtain estimates for :

- the rate of molecular evolution
- the date of the most recent common ancestor
- the phylogenetic relationships with measures of statistical support.

The following software will be used in this tutorial:

- **BEAST** - this package contains the BEAST program, BEAUti, DensiTree, TreeAnnotator and other utility programs. This tutorial is written for BEAST v2.0, which has support for multiple partitions. It is available for download from `http://beast2.cs.auckland.ac.nz/`.
- **Tracer** - this program is used to explore the output of BEAST (and other Bayesian MCMC programs). It graphically and quantitively summarizes the distributions of continuous parameters and provides diagnostic information. At the time of writing, the current version is v1.5. It is available for download from `http://beast.bio.ed.ac.uk/`.
- **FigTree** - this is an application for displaying and printing molecular phylogenies, in particular those obtained using BEAST. At the time of writing, the current version is v1.3.1. It is available for download from `http://tree.bio.ed.ac.uk/`.

## The NEXUS alignment

The data is in a file called `RSV2.nex` and you can find it in the `examples/nexus` directory in the directory where BEAST was installed. This file contains an alignment of 129 sequences from the G gene of RSVA virus, 629 nucleotides in length. Import this alignment into BEAUti. Because this is a protein-coding gene we are going to split the alignment into three partitions representing each of the three codon positions. To do this we will click the **Split** button at the bottom of the **Partitions** panel and then select the "1 + 2 + 3 frame 3" from the drop-down menu. This signifies that the first full codon starts at the third nucleotide in the alignment. This will create three rows in the partitions panel. You will have to re-link the tree and clock models across the three partitions (and name them "tree" and "clock" respectively) before continuing to the next step.

The partition panel should now look something like this:

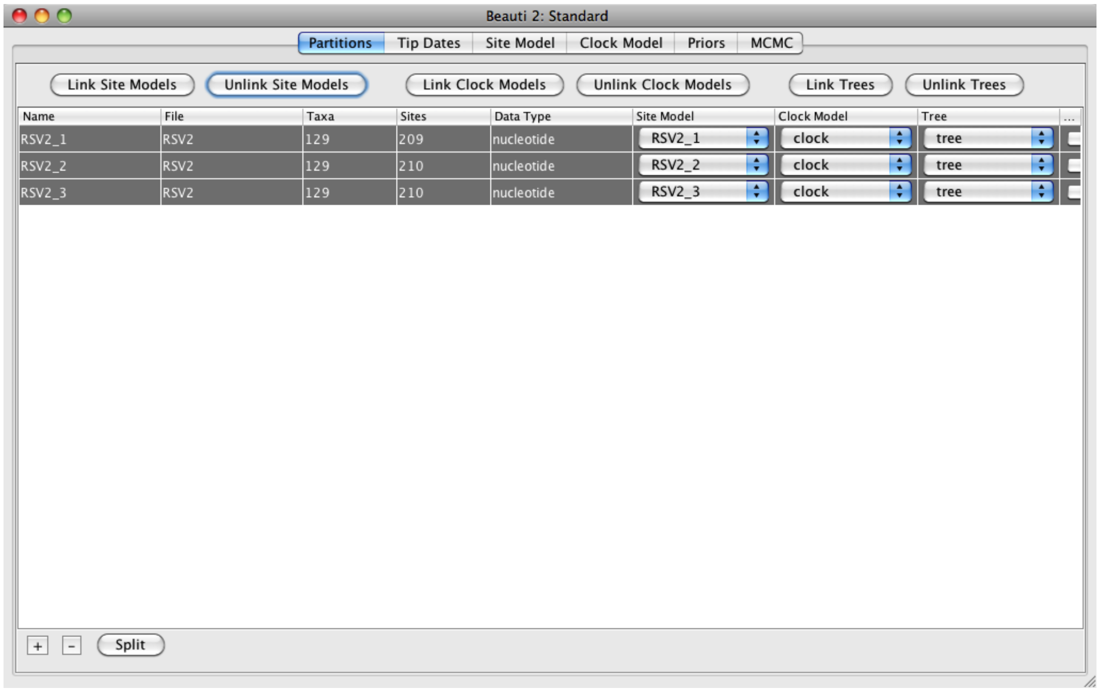


By default all the taxa are assumed to have a date of zero (i.e. the sequences are assumed to be sampled at the same time). In this case, the RSVA sequences have been sampled at various dates going back to the 1950s. The actual year of sampling is given in the name of each taxon and we could simply edit the value in the Date column of the table to reflect these. However, if the taxa names contain the calibration information, then a convenient way to specify the dates of the sequences in BEAUti is to click the checkbox **Use tip dates** and then use the **Guess** button at the top of the **Tip Dates** panel. Clicking this will make a dialog box appear.

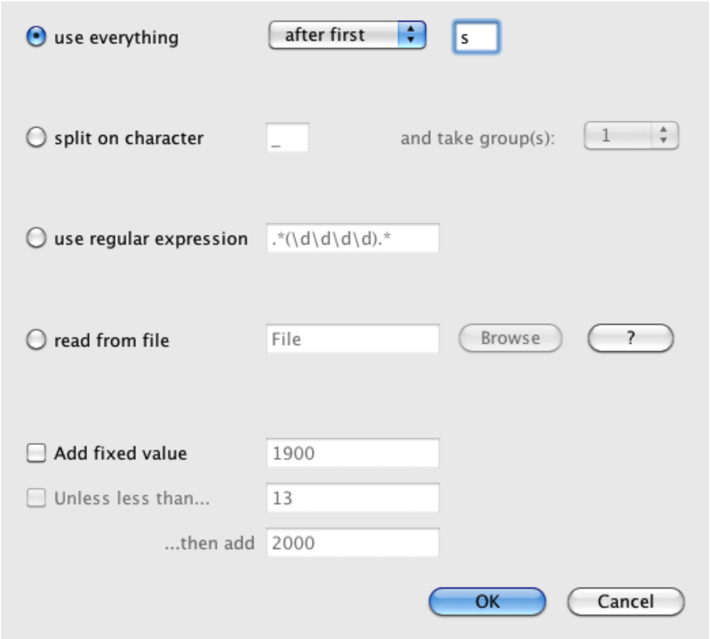


Select the option to *use everything*, choose "after last" from from drop-down box and type's' into the corresponding text box. This will extract the trailing numbers from the taxon names after the last little 's', which are interpreted as the year (in this case since 1900) that the sample was isolated.

The dates panel should now look something like this:

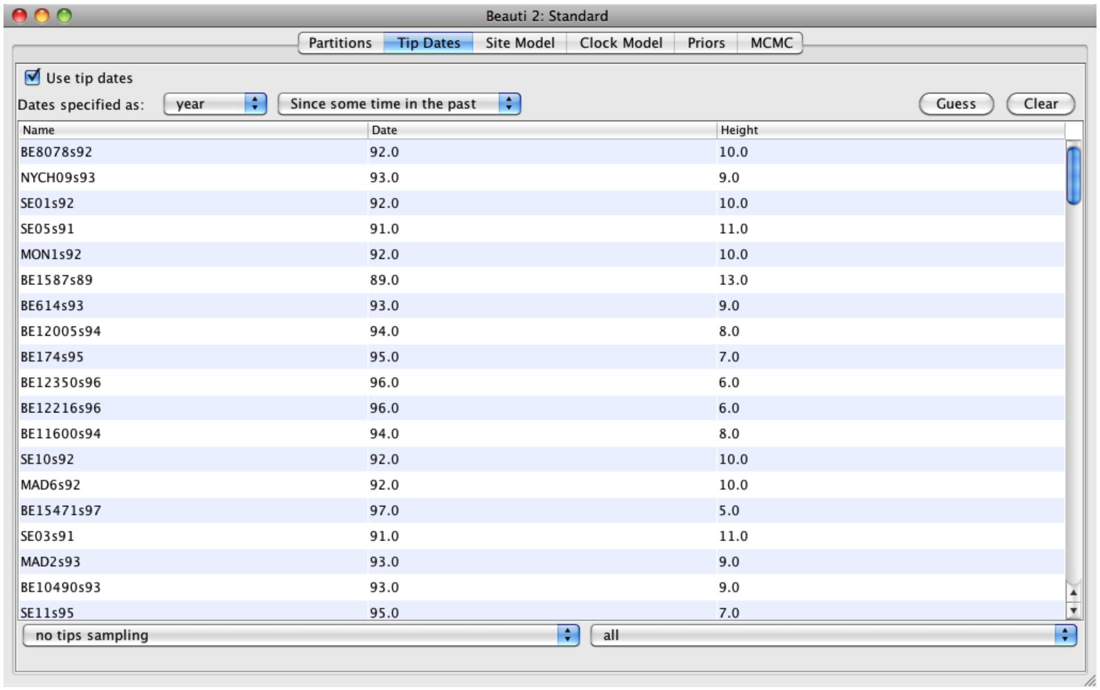

## Setting the substitution model

We will use the HKY model with empirical base frequencies for all three partitions. To do this first link the site partitions and then choose HKY and Empirical from the Subst Model and Frequencies drop-boxes. Also check the estimate box for the Mutation Rate and finally check the "Fix mean mutation rate" box. Then go back to Partitions panel and unlink the partitions.

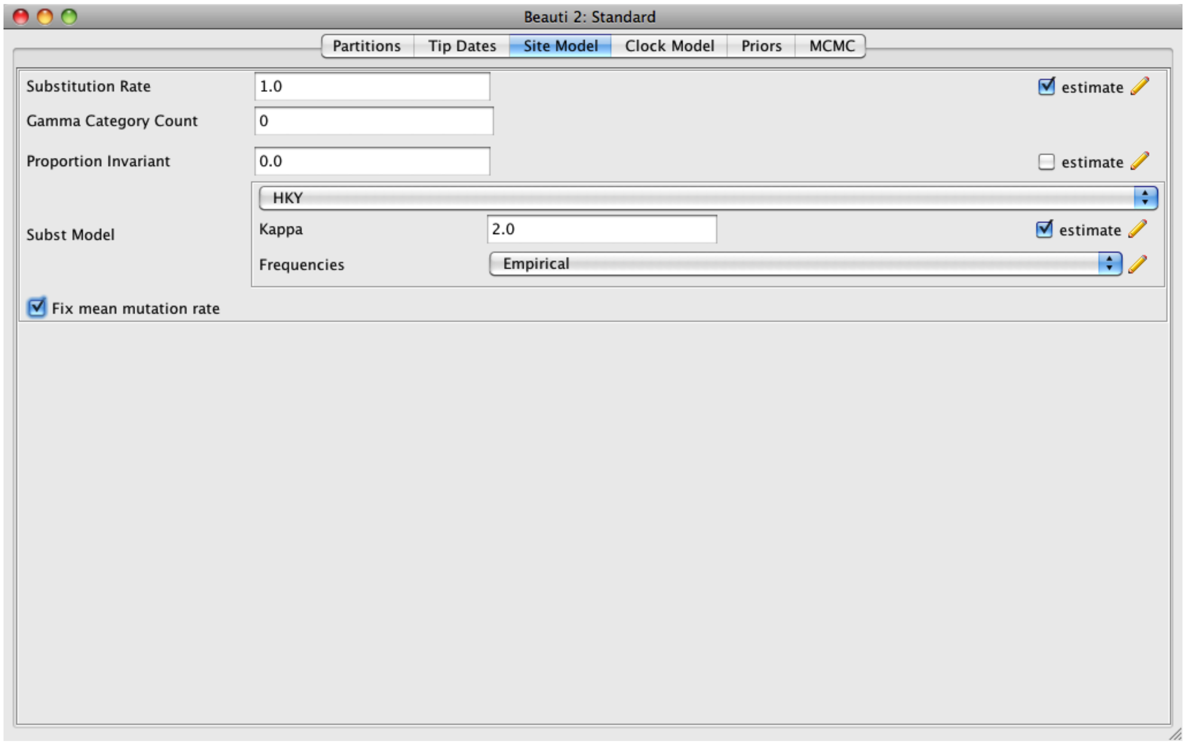


### 1.0.1 Priors

To set up the priors, select the "Priors" tab. Choose "Coalescent Constant Population" for the tree prior. Set the prior on the clockRate parameter to a log-normal with $M = -5$ and $S = 1.25$.

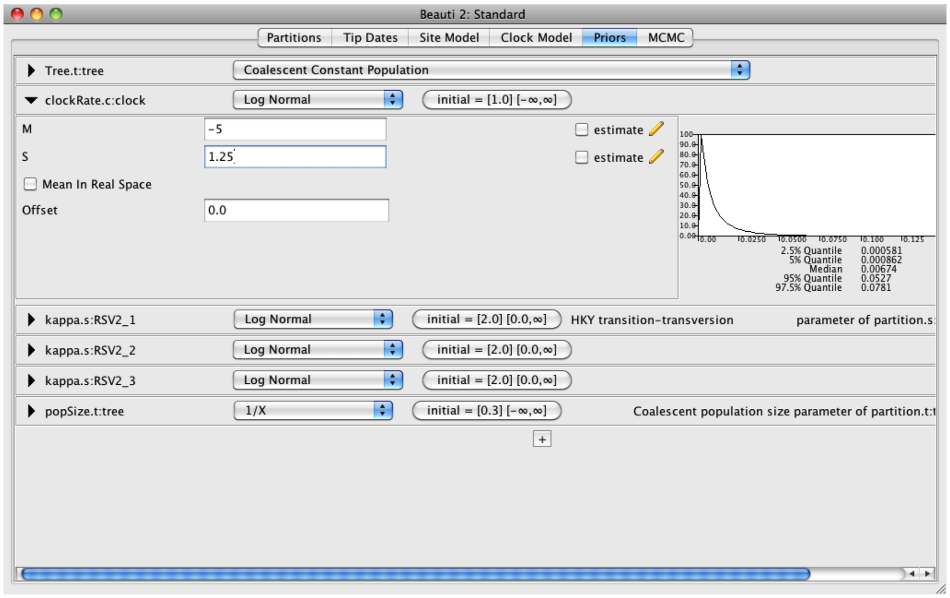


## 1.1 Setting the MCMC options

For this dataset let's initially set the chain length to 2,000,000 as this will run reasonably quickly on most modern computers. Set the sampling frequencies for the screen to to 1000, the trace log file to 400 and the trees file to 400.

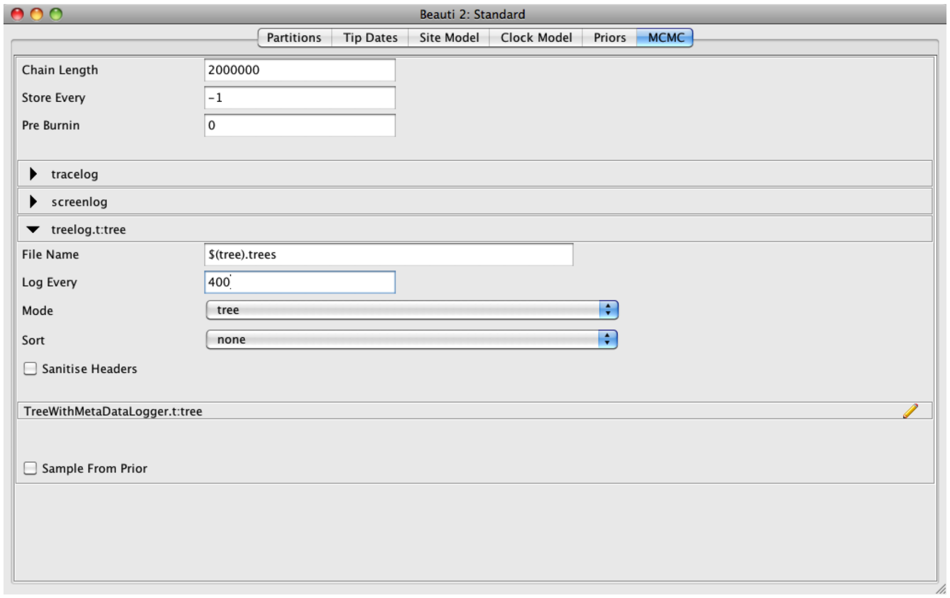


## Running BEAST

Save the BEAST file (e.g. `RSV2.xml`) and run it in BEAST.

## Analysing the BEAST output

Note that the effective sample sizes (ESSs) for many of the logged quantities are small (ESSs less than 100 will be highlighted in red by Tracer). This is not good. A low ESS means that the trace contains a lot of correlated samples and thus may not represent the posterior distribution well. In the bottom right of the window is a frequency plot of the samples which is expected given the low ESSs is extremely rough.

If we select the tab on the right-hand-side labelled 'Trace' we can view the raw trace, that is, the sampled values against the step in the MCMC chain.

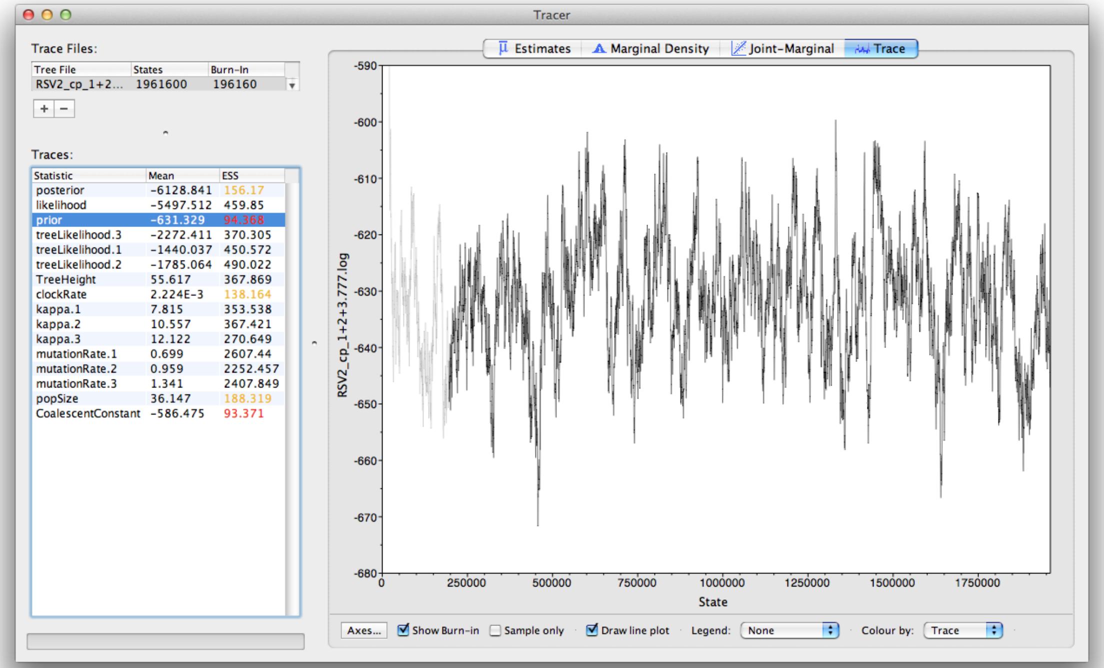


Here you can see how the samples are correlated. There are 5000 samples in the trace (we ran the MCMC for 2,000,000 steps sampling every 400) but adjacent samples often tend to have similar values. The ESS for the absolute rate of evolution (clockRate) is about 138 so we are only getting 1 independent sample to every $36 = 5000/138$ actual samples). With a short run such as this one, it may also be the case that the default burn-in of 10% of the chain length is inadequate. Not excluding enough of the start of the chain as burn-in will render estimates of ESS unreliable.

The simple response to this situation is that we need to run the chain for longer. Given the lowest ESS (for the constant coalescent prior) is 93, it would suggest that we have to run the chain for at least twice the length to get reasonable ESSs that are >200. However it would be better to aim higher so lets go for a chain length of 10,000,000. Go back to the **MCMC** options section in BEAUti, and create a new BEAST XML file with a longer chain length. Now run BEAST and load the new log file into Tracer (you can leave the old one loaded for comparison).

Click on the Trace tab and look at the raw trace plot.

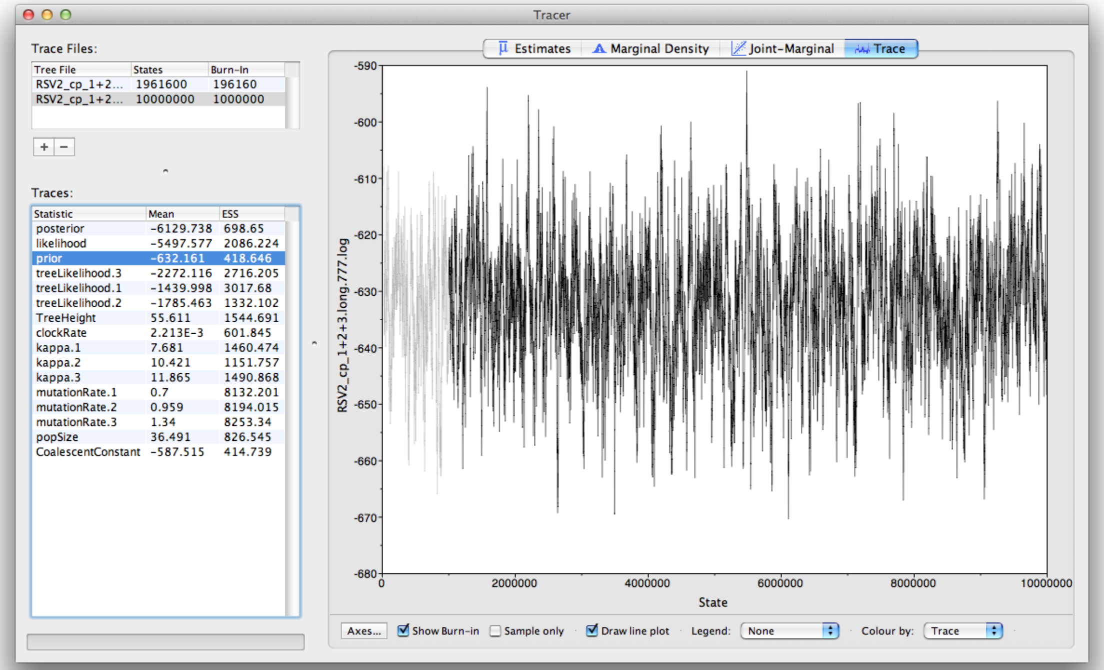


Again we have chosen options that produce 5000 samples and with an ESS of about 419 there is still auto-correlation between the samples but >400 effectively independent samples will now provide a very good estimate of the posterior distribution. There are no obvious trends in the plot which would suggest that the MCMC has not yet converged, and there are no significant long range fluctuations in the trace which would suggest poor mixing.

As we are satisfied with the mixing we can now move on to one of the parameters of interest: substitution rate. Select `clockRate` in the left-hand table. This is the average substitution rate across all sites in the alignment. Now choose the density plot by selecting the tab labeled *Marginal Density*. This shows a plot of the marginal posterior probability density of this parameter. You should see a plot similar to this:

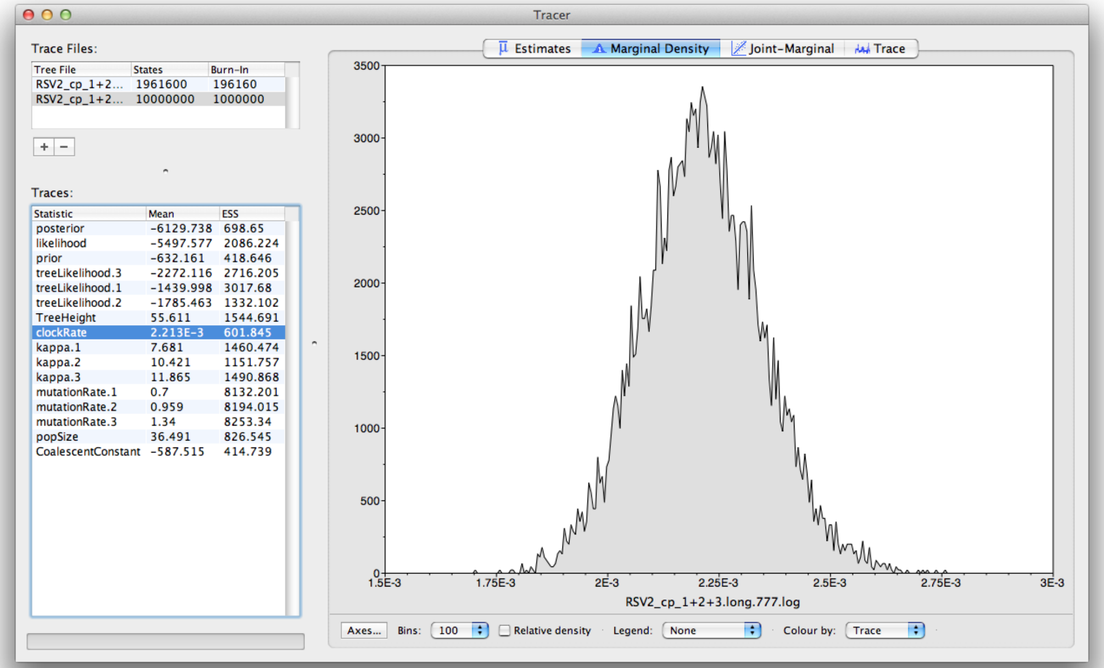


As you can see the posterior probability density is roughly bell-shaped. There is some sampling noise which would be reduced if we ran the chain for longer or sampled more often but we already have a good estimate of the mean and HPD interval. You can overlay the density plots of multiple traces in order to compare them (it is up to the user to determine whether they are comparable on the the same axis or not). Select the relative substitution rates for all three codon positions in the table to the left (labelled `mutationRate.1`, `mutationRate.2` and `mutationRate.3`). You will now see the posterior probability densities for the relative substitution rate at all three codon positions overlaid:

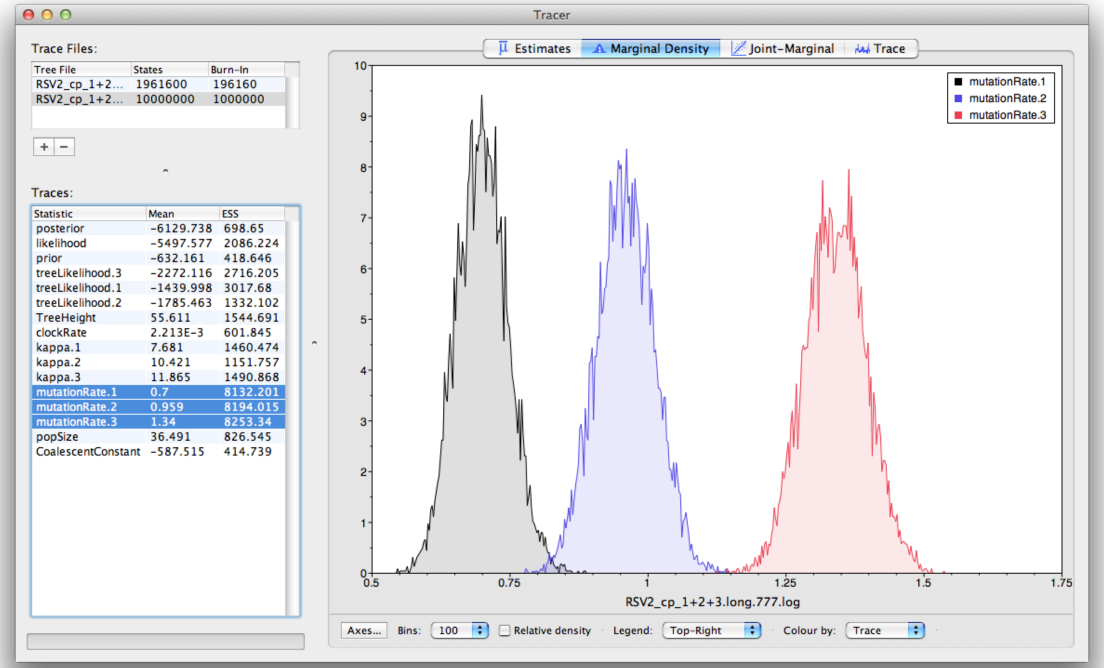


## Summarizing the trees

Use the program TreeAnnotator to summarize the tree and view the results in Figtree (Figure 1).

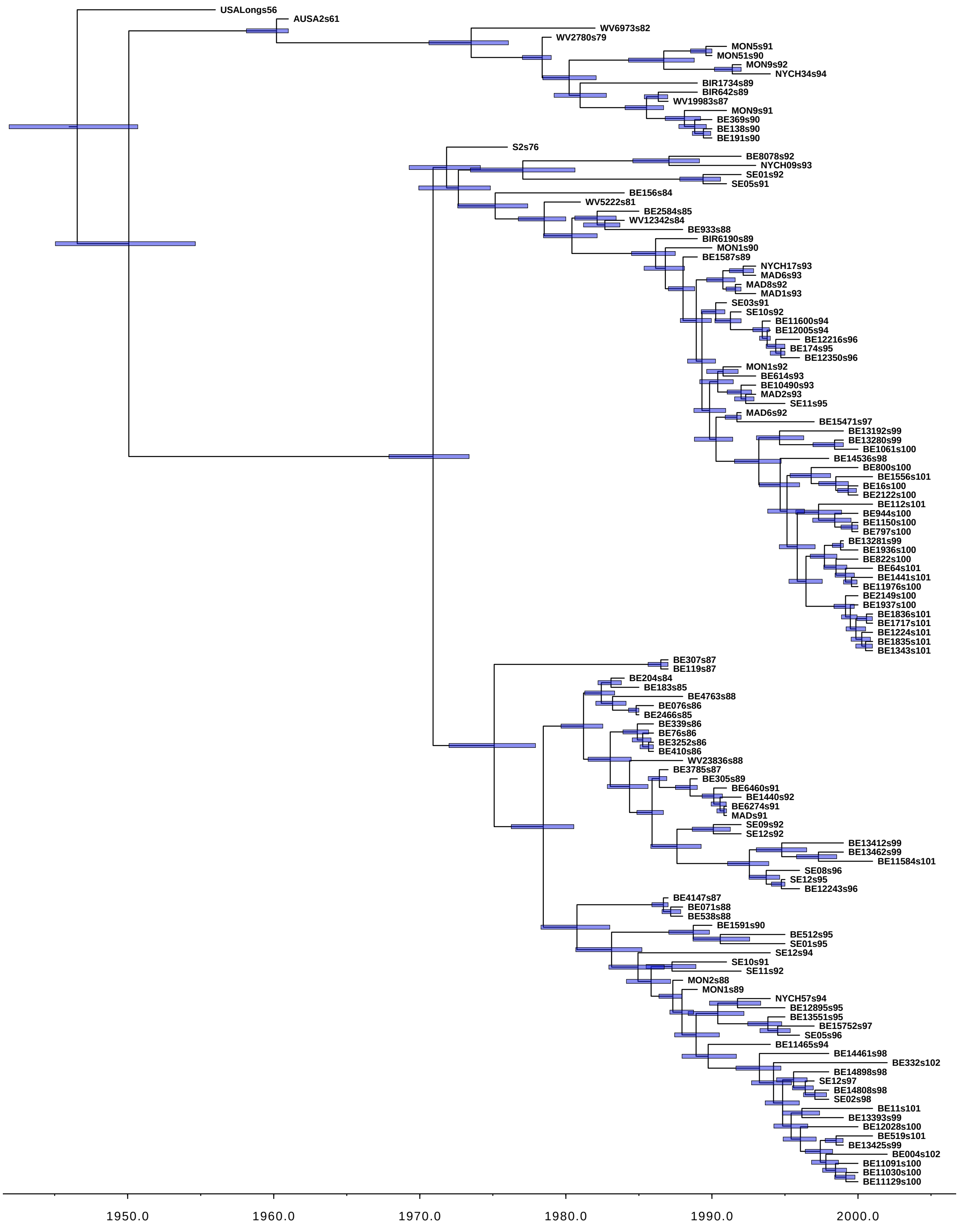


Figure 1: The Maximum clade credibility tree for the G gene of 129 RSVA-2 viral samples.

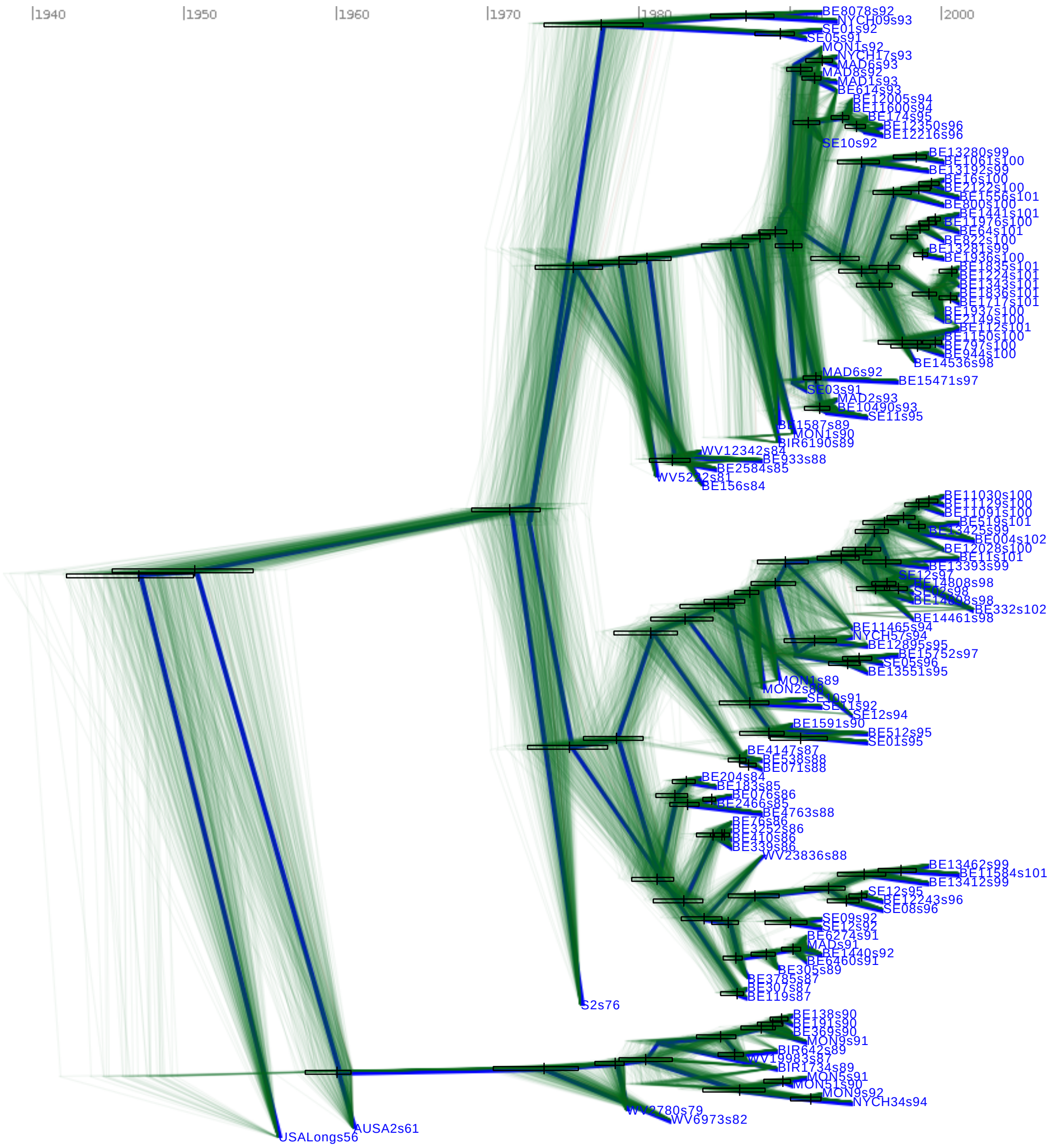


DensiTree with clade height bars for clades with over 50% support. Root canal tree represents maximum clade credibility tree.

## Questions

*In what year did the common ancestor of all RSVA viruses sampled live? What is the 95% HPD?*

# References

[1] Kalina T Zlateva, Philippe Lemey, Elien Moës, Anne-Mieke Vandamme, and Marc Van Ranst, *Genetic variability and molecular evolution of the human respiratory syncytial virus subgroup b attachment g protein*, J Virol **79** (2005), no. 14, 9157–67.

[2] Kalina T Zlateva, Philippe Lemey, Anne-Mieke Vandamme, and Marc Van Ranst, *Molecular evolution and circulation patterns of human respiratory syncytial virus subgroup a: positively selected sites in the attachment g glycoprotein*, J Virol **78** (2004), no. 9, 4675–83.
