# Supplementary material for: BEAST 2: A Software Platform for Bayesian Evolutionary Analysis
Source: PLoS Comput Biol. 2014 Apr 10;10(4):e1003537. doi: 10.1371/journal.pcbi.1003537 (PMC3985171; doi:10.1371/journal.pcbi.1003537)
Supplement: Text S3 — Tutorial for multi-species coalescent with *BEAST. (PDF) [file pcbi.1003537.s006.pdf]

*BEAST in BEAST 2.0
Estimating Species Trees from Multilocus Data

Joseph Heled, Remco Bouckaert, Alexei J Drummond and Walter Xie

December 3, 2013

1 Introduction

In this tutorial we describe a full Bayesian framework for species tree estimation. The statistical methodology described in this tutorial is known by the acronym *BEAST (pronounced "star beast") [2].

You will need the following software at your disposal:

- **BEAST** - this package contains the BEAST program, BEAUti, TreeAnnotator and other utility programs. This tutorial is written for BEAST v2.0, which has support for multiple partitions. It is available for download from
`http://beast2.cs.auckland.ac.nz/`.
- **Tracer** - this program is used to explore the output of BEAST (and other Bayesian MCMC programs). It graphically and quantitively summarizes the distributions of continuous parameters and provides diagnostic information. At the time of writing, the current version is v1.5. It is available for download from `http://beast.bio.ed.ac.uk/`.
- **FigTree** - this is an application for displaying and printing molecular phylogenies, in particular those obtained using BEAST. At the time of writing, the current version is v1.3.1. It is available for download from `http://tree.bio.ed.ac.uk/`.

2 *BEAST

This tutorial will guide you through the analysis of three loci sampled from 26 individuals representing nine species of pocket gophers. This is a subset of previous published data [1]. The objective of this tutorial is to estimate the species tree that is most probable given the multi-individual multi-locus sequence data. The species tree has nine taxa, whereas each gene tree has 26 taxa. *BEAST 2 will co-estimate three gene trees embedded in a shared species tree [2, for details].

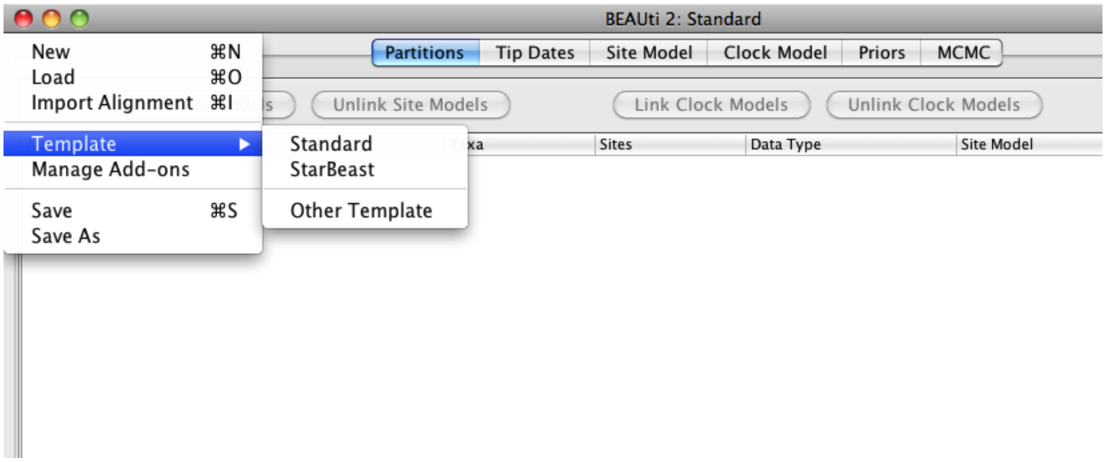

Figure 1: Select a new template in BEAUti.

The first step will be to convert a NEXUS file with a DATA or CHARACTERS block into a BEAST XML input file. This is done using the program BEAUti (Bayesian Evolutionary Analysis Utility). This is a user-friendly program for setting the evolutionary model and options for the MCMC analysis. The second step is to actually run BEAST using the input file that contains the data, model and settings. The final step is to explore the output of BEAST in order to diagnose problems and to summarize the results.

BEAUti

Run BEAUti by double clicking on its icon.

Set up BEAUti for *BEAST

*BEAST uses a different template from the standard. This means that to use BEAUti for *BEAST, the first thing to do is change the template. Choose the File/Templates/StarBeast item. When changing a template, BEAUti deletes all previously imported data and start with a new empty template. So, if you already loaded some data, a warning message pops up indicating that this data will be lost if you switch templates.

Loading the NEXUS file

*BEAST is a multi-individual, multi-locus method method. The data for each locus is stored as one alignment in its own NEXUS file. Taxa names in each alignment have to be unique, but duplicates across alignments are fine.

To load a NEXUS format alignment, simply select the `Import Alignment` option from the File menu:

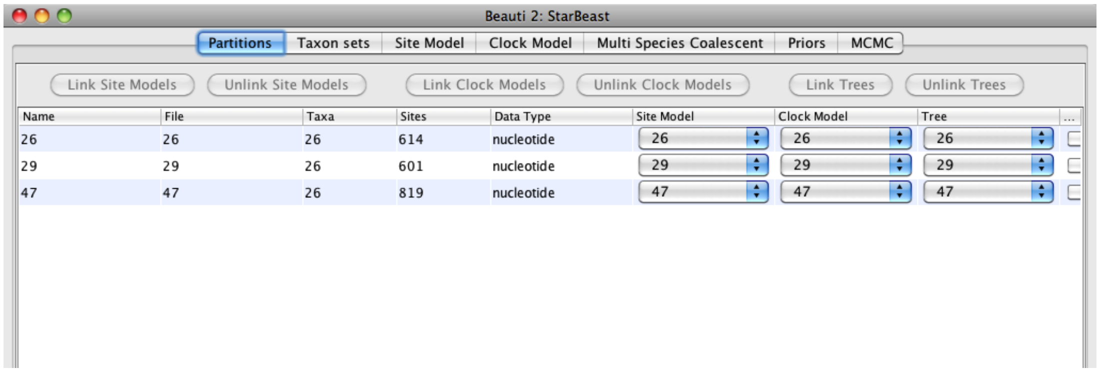

Figure 2: Data partition panel after loading alignments.

Select three files called `26.nex, 29.nex, 47.nex` by holding `shift` key. You can find the files in the `examples/nexus` directory in the directory where BEAST was installed. Each file contains an alignment of sequences of from an independent locus. The `26.nex` looks like this (content has been truncated):

```
#NEXUS
[TBO26oLong]
BEGIN DATA;
DIMENSIONS  NTAX =26 NCHAR=614;
FORMAT DATATYPE = DNA GAP = - MISSING = ?;
MATRIX
Orthogeomys_heterodus         ATTCTAGGCAAAAAGAGCAATGC ...
Thomomys_bottae_awahnee_a     ????????????????????ATGCTG ...
Thomomys_bottae_awahnee_b     ????????????????????ATGCTG ...
Thomomys_bottae_xerophilus    ????????????????????ATGCTG ...
Thomomys_bottae_cactophilus   ????????????????AGCAATGCT ...

         ... ...

;
END;
```

Once loaded, the three partitions are displayed in the main panel. You can double click any alignment (partition) to show its detail.

For multi-locus analyses, BEAST can link or unlink substitutions models across the loci by clicking buttons on the top of **Partitions** panel. The default of *BEAST is unlinking all models: substitution model, clock model, and tree models. Note that you should only unlink the tree model across data partitions that are actually genetically unlinked. For example, in most organisms all the

mitochondrial genes are effectively linked due to a lack of recombination and they should be set up to use the same tree model in a *BEAST analysis.

Import trait(s) from a mapping file to fire *BEAST

Each taxon in a *BEAST analysis is associated with a species. Typically the species name is already embedded inside the taxon. The species name should be easy to extract; place it either at the beginning or the end, separated by a "special" character which does not appear in names. For example, `aria_334259, coast_343436` (using an underscore) or `10x017b.wrussia, 2x305b.eastis` (using a dot).

We need to tell BEAUti somehow which lineages in the alignments go with taxa in the species tree. Select the Taxon Set panel, and a list of taxa from the alignments is shown together with a default guess by BEAUti. In this case, the guess is not very good, so we want to change this. You can manually change each of the entries in the table, or press the guess button and a dialog is shown where you can choose from several ways to try to detect the taxon from the name of the lineages, or have a mapping stored in a file. In this case, splitting the name on the underscore character ('_') and selecting the second group will give us the mapping that we need.

Alternatively, the mapping can be read from a trait file. A proper trait file is tab delimited. The first row is always `traits` followed by the keyword `species` in the second column and separated by tab. The rest of the rows map each individual taxon name to a species name: the taxon name in the first column and species name in the second column separated by tab. For example:

```
traits species
taxon1 speciesA
taxon2 speciesA
taxon3 speciesB
... ...
```

Setting the substitution model

The next thing to do is to click on the **Site Model** tab at the top of the main window. This will reveal the evolutionary model settings for BEAST. Exactly which options appear depend on whether the data are nucleotides, or amino acids, or binary data, or general data. The settings that will appear after loading the data set will be the default values so we need to make some changes.

Most of the models should be familiar to you. For this analysis, we will select each substitution model listed on the left side in turn to make the following change: select HKY for substitution model and **Empirical** for the **Frequencies**.

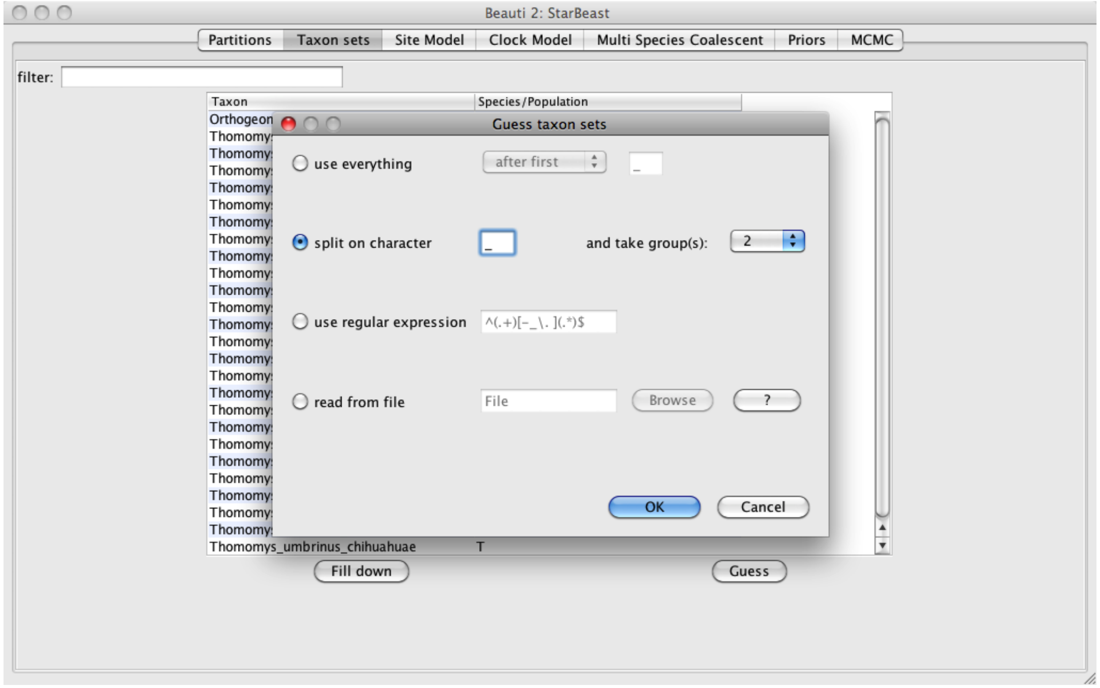

Figure 3: Selecting taxon sets in BEAUti using the guess dialog from the taxon set panel.

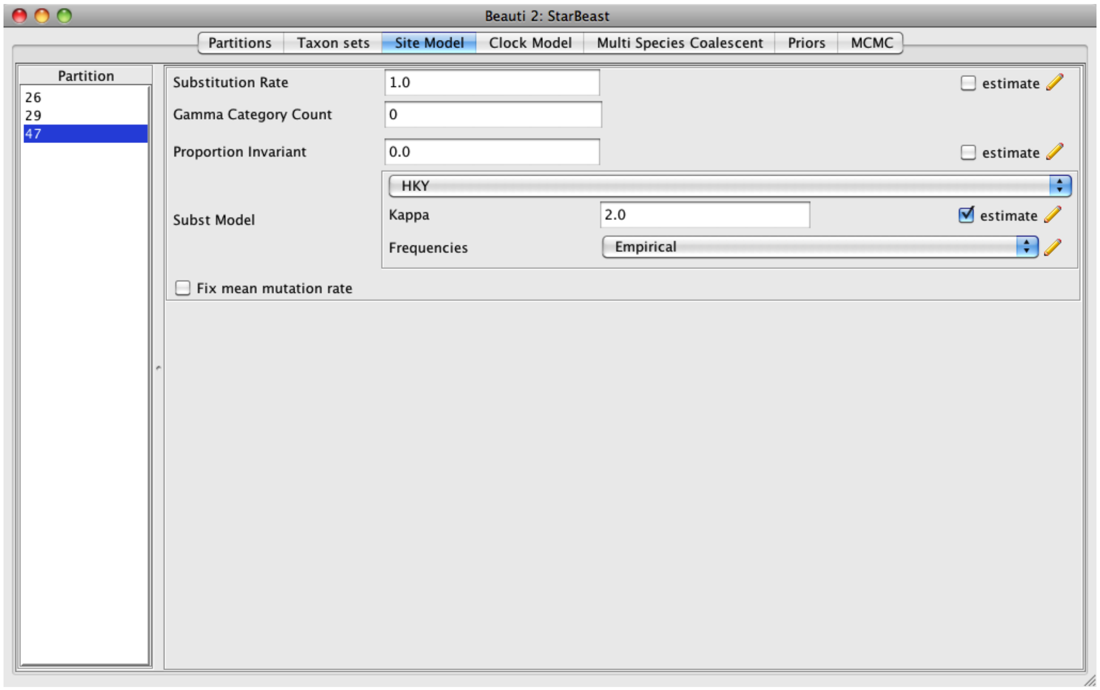

Figure 4: Setting up substitution and site models for the gopher alignments.

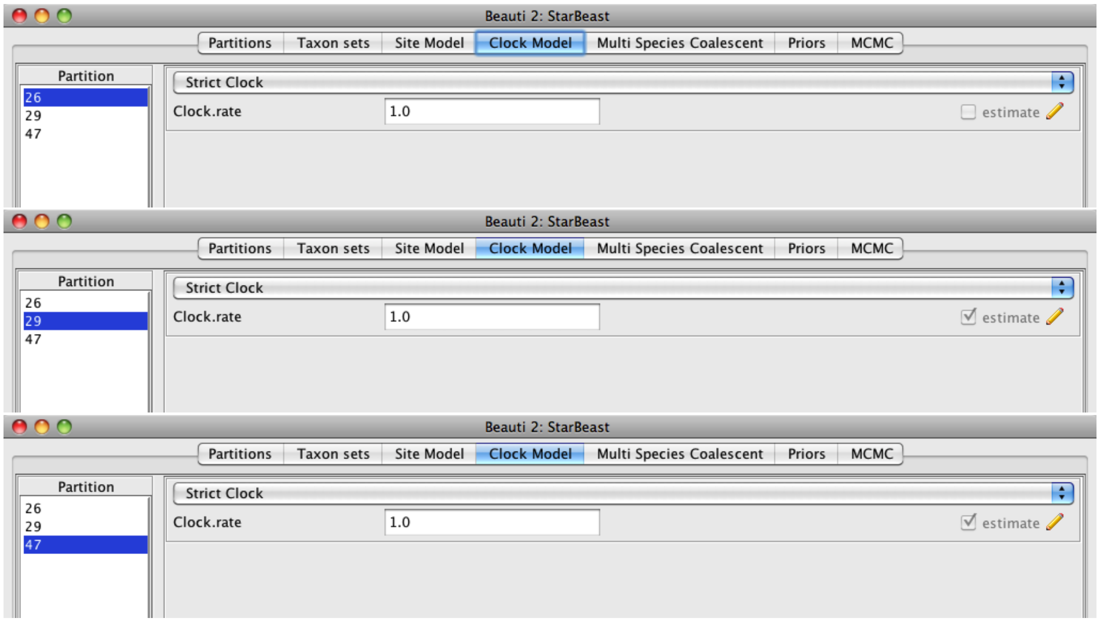

Figure 5: Setting up clock models for the gopher alignments.

Setting the clock model

Second, click on the **Clock Models** tab at the top of the main window. In this analysis, we use the **Strict Clock** molecular clock model as default. Your model options should now look like this:

The **Estimate** check box is unchecked for the first clock model and checked for the rest clock models, because we wish to estimate the substitution rate of each subsequent locus relative to the first locus whose rate is fixed to 1.0.

Multi Species Coalescent

The **Multi Species Coalescent** panel allows settings to the multi species coalescent model to be specified for each tree. *BEAST has a different tree prior panel where users can only configure the species tree prior not gene tree priors (which are automatically specified by the multispecies coalescent). Currently, we have two species tree priors: **Yule Process** and **Birth-Death Process**; and three population size models: **Piecewise linear and constant root**, **Piecewise linear**, and **Piecewise constant**. In this analysis, we use piecewise linear and constant root.

The **Ploidy** item determines the type of sequence (mitochondrial, nuclear, X, Y). This matters since different modes of inheritance gives rise to different effective population sizes. In this analysis, we simply use a random starting tree.

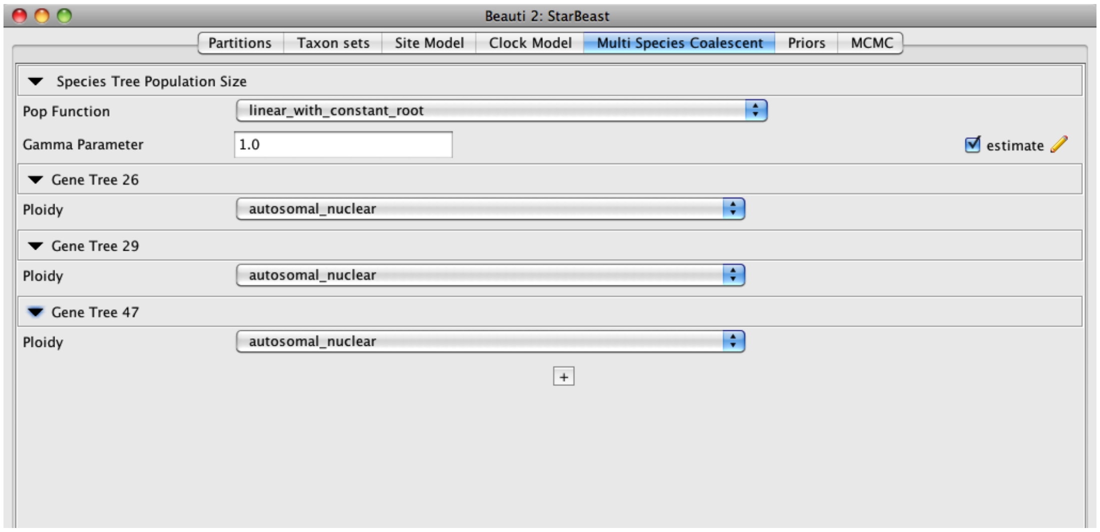

Figure 6: Setting up multi species coalescent parameters.

Priors and Operators

The **Priors** panel allows priors to be specified for each parameter in the model. The **Operators** panel (hidden) is used to configure technical settings that affect the efficiency of the MCMC program. We leave these two panels unchanged in this analysis.

Setting the MCMC options

The next tab, **MCMC**, provides more general settings to control the length of the MCMC and the file names.

Firstly we have the **Length of chain**. This is the number of steps the MCMC will make in the chain before finishing. The appropriate length of the chain depends on the size of the data set, the complexity of the model and the accuracy of the answer required. The default value of 10,000,000 is entirely arbitrary and should be adjusted according to the size of your data set. For this data set let's keep the chain length at 10,000,000 as this will run reasonably quickly on most modern computers (less than 20 minutes).

The next options specify how often the parameter values in the Markov chain should be displayed on the screen and recorded in the log file. The screen output is simply for monitoring the programs progress so can be set to any value (although if set too small, the sheer quantity of information being displayed on the screen will actually slow the program down). For the log file, the value should be set relative to the total length of the chain. Sampling too often will result in very large files with little extra benefit in terms of the precision of

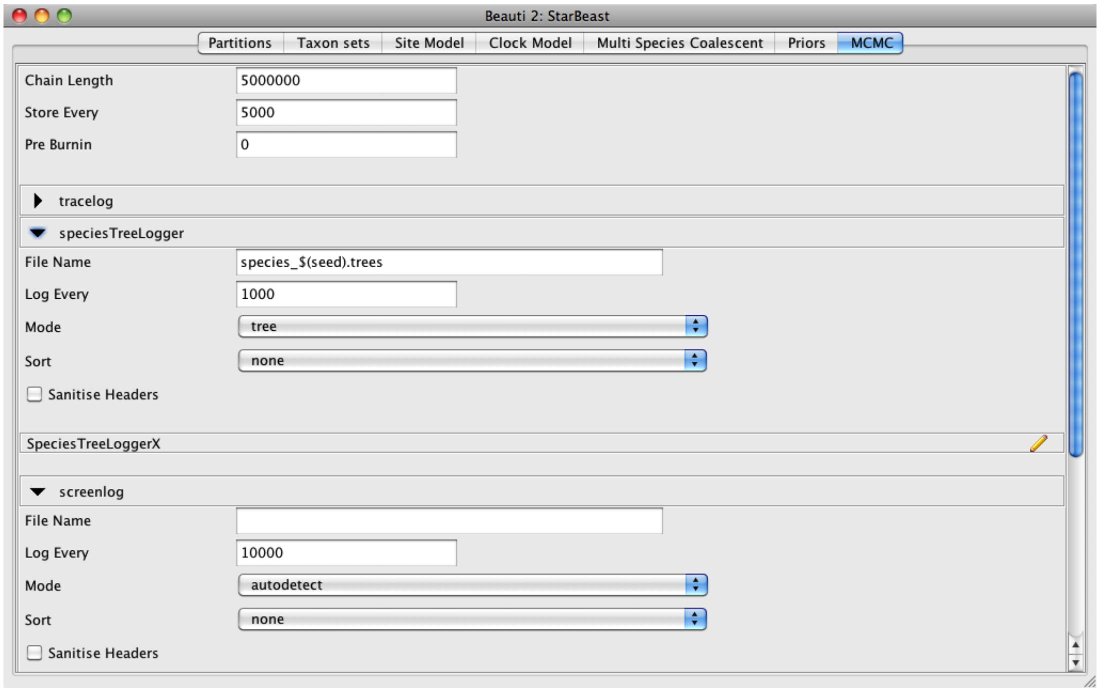

Figure 7: Setting up the MCMC paremeters.

the analysis. Sample too infrequently and the log file will not contain much information about the distributions of the parameters. You probably want to aim to store no more than 10,000 samples so this should be set to no less than chain length / 10,000.

For this exercise we will set the screen log to 10000 and the trace log to 1000. The final two options give the file names of the log files for the sampled parameters and the trees. These will be set to a default based on the name of the imported NEXUS file.

If you are using windows then we suggest you add the suffix `.txt` to both of these (so, `gopher.log.txt` and `gopher.trees.txt`) so that Windows recognizes these as text files.

Generating the BEAST XML file

We are now ready to create the BEAST XML file. To do this, either select the **File/Save** or **File/Save As** option from the **File** menu. Check the default priors setting and click **Continue**. Save the file with an appropriate name (we usually end the filename with `.xml`, i.e., `gopher.xml`). We are now ready to run the file through BEAST.

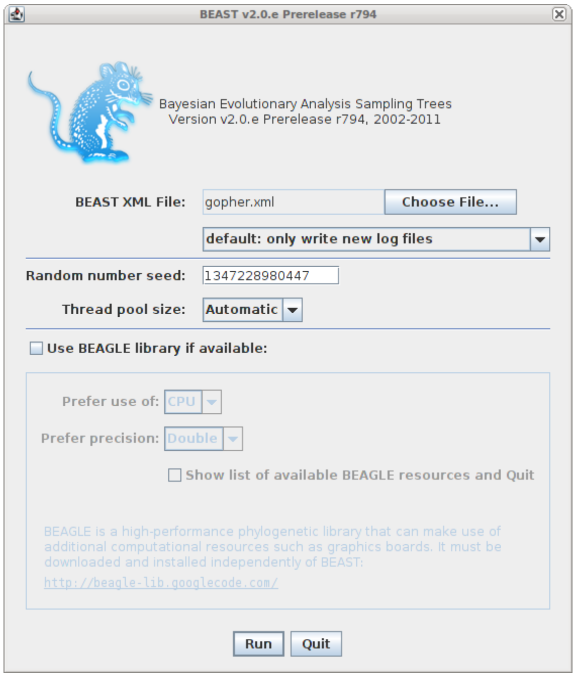

Figure 8: Launching BEAST.

Running BEAST

Now run BEAST and when it asks for an input file, provide your newly created XML file as input by click **Choose File ...**, and then click **Run**.

BEAST will then run until it has finished reporting information to the screen. The actual results files are saved to the disk in the same location as your input file. The output to the screen will look something like this:

```
          BEAST v2.0.e Prerelease r794, 2002-2011
       Bayesian Evolutionary Analysis Sampling Trees
                 Designed and developed by
Remco Bouckaert, Alexei J. Drummond, Andrew Rambaut and Marc A. Suchard

               Department of Computer Science
                   University of Auckland
                  remco@cs.auckland.ac.nz
                  alexei@cs.auckland.ac.nz

             Institute of Evolutionary Biology
                  University of Edinburgh
                     a.rambaut@ed.ac.uk

              David Geffen School of Medicine
           University of California, Los Angeles
                     msuchard@ucla.edu

                Downloads, Help & Resources:
               http://beast2.cs.auckland.ac.nz
```

```
Source code distributed under the GNU Lesser General Public License:
               http://code.google.com/p/beast2

                     BEAST developers:
Alex Alekseyenko, Trevor Bedford, Erik Bloomquist, Joseph Heled,
Sebastian Hoehna, Denise Kuehnert, Philippe Lemey, Wai Lok Sibon Li,
Gerton Lunter, Sidney Markowitz, Vladimir Minin, Michael Defoin Platel,
           Oliver Pybus, Chieh-Hsi Wu, Walter Xie

                         Thanks to:
     Roald Forsberg, Beth Shapiro and Korbinian Strimmer

... ...


        4990000     -3820.9967        91.3        -4288.0542        19.8887 57s/Msamples
        5000000     -3810.3221        91.6        -4299.7884        23.2278 57s/Msamples
Operator                                                              Tuning #accept #reject #total
acceptance rate
beast.evolution.operators.NodeReheight_Reheight                              377607 911781 1289388
0.293
beast.evolution.operators.ScaleOperator_popSizeScaler                 0.182  18180 50730 68910
0.264
beast.evolution.operators.UpDownOperator_updown.all                   0.486  67603 207017 274620
0.246
beast.evolution.operators.ScaleOperator_YuleBirthRateScaler.Species   0.228  12288 29165 41453
0.296
beast.evolution.operators.ScaleOperator_popMeanScale                  0.491  11242 30098 41340
0.272
beast.evolution.operators.ScaleOperator_treeScaler.t:26               0.777  7496 33737 41233
0.182
beast.evolution.operators.ScaleOperator_treeRootScaler.t:26           0.423  8592 32825 41417
0.207
beast.evolution.operators.Uniform_UniformOperator.t:26                       231886 180217 412103
0.563
beast.evolution.operators.SubtreeSlide_SubtreeSlide.t:26              0.481  391 205278 205669
0.002 Try decreasing size to about 0.241
beast.evolution.operators.Exchange_narrow.t:26                               93812 112277 206089
0.455
beast.evolution.operators.Exchange_wide.t:26                                 1006 40176 41182
0.024
beast.evolution.operators.WilsonBalding_WilsonBalding.t:26                   1443 39692 41135
0.035
beast.evolution.operators.ScaleOperator_StrictClockRateScaler.c:29    0.481  10889 30421 41310
0.264
beast.evolution.operators.ScaleOperator_treeScaler.t:29               0.764  6915 34490 41405
0.167
beast.evolution.operators.ScaleOperator_treeRootScaler.t:29           0.418  10155 30810 40965
0.248
beast.evolution.operators.Uniform_UniformOperator.t:29                       237464 173721 411185
0.578
beast.evolution.operators.SubtreeSlide_SubtreeSlide.t:29              0.346  448 205862 206310
0.002 Try decreasing size to about 0.173
beast.evolution.operators.Exchange_narrow.t:29                               95926 109390 205316
0.467
beast.evolution.operators.Exchange_wide.t:29                                 1772 39232 41004
0.043
beast.evolution.operators.WilsonBalding_WilsonBalding.t:29                   2025 39408 41433
0.049
beast.evolution.operators.UpDownOperator_updown.29                    0.803  9954 31223 41177
0.242
beast.evolution.operators.UpDownOperator_strictClockUpDownOperator.c:290.788  8922 32105 41027
0.217
beast.evolution.operators.ScaleOperator_StrictClockRateScaler.c:47    0.556  11000 30234 41234
0.267
beast.evolution.operators.ScaleOperator_treeScaler.t:47               0.746  7268 33584 40852
0.178
```

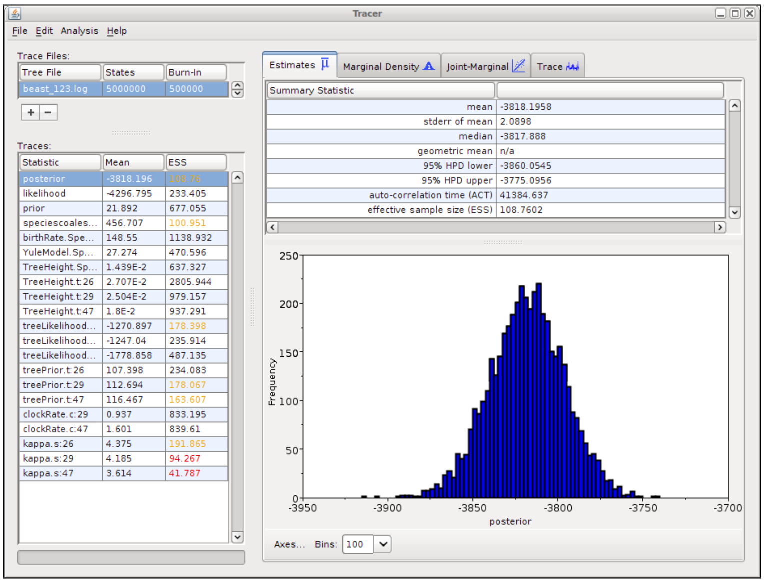

Figure 9: Tracer with the gopher data.

```
beast.evolution.operators.ScaleOperator_treeRootScaler.t:47          0.515  7435 33814 41249
0.18
beast.evolution.operators.Uniform_UniformOperator.t:47                      223624 188613 412237
0.542
beast.evolution.operators.SubtreeSlide_SubtreeSlide.t:47             0.506  228 205368 205596
0.001 Try decreasing size to about 0.253
beast.evolution.operators.Exchange_narrow.t:47                              74248 131396 205644
0.361
beast.evolution.operators.Exchange_wide.t:47                                582 40815 41397 0.014
beast.evolution.operators.WilsonBalding_WilsonBalding.t:47                  754 40484 41238 0.018
beast.evolution.operators.UpDownOperator_updown.47                   0.774  10815 30475 41290
0.262
beast.evolution.operators.UpDownOperator_strictClockUpDownOperator.c:470.788  11351 29559 40910
0.277
beast.evolution.operators.ScaleOperator_KappaScaler.s:26             0.339  407 999 1406 0.289
beast.evolution.operators.ScaleOperator_KappaScaler.s:29             0.277  330 1091 1421 0.232
beast.evolution.operators.ScaleOperator_KappaScaler.s:47             0.329  333 1005 1338 0.249
beast.evolution.operators.ScaleOperator_popSizeTopScaler             0.166  18005 50513 68518
0.263
Total calculation time: 291.267 seconds
End likelihood: -3810.322148438054
```

Analyzing the results

Run the program called **Tracer** to analyze the output of BEAST. When the main window has opened, choose **Import Trace File...** from the **File** menu and select the file that BEAST has created called `gopher.log`. You should now see a window like in Figure 9.

Remember that MCMC is a stochastic algorithm so the actual numbers will not be exactly the same.

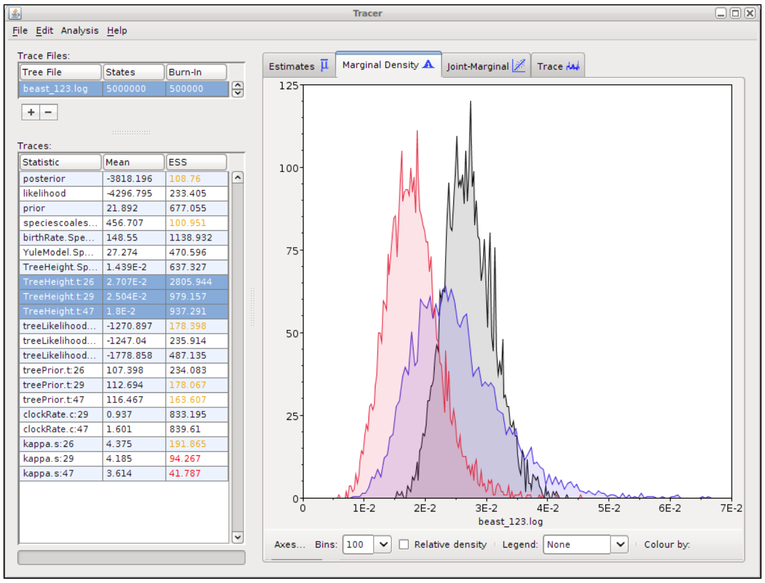

Figure 10: Tracer showing the root heights of the lineage trees.

On the left hand side is a list of the different quantities that BEAST has logged. There are traces for the posterior (this is the log of the product of the tree likelihood and the prior probabilities), and the continuous parameters. Selecting a trace on the left brings up analyses for this trace on the right hand side depending on tab that is selected. When first opened, the 'posterior' trace is selected and various statistics of this trace are shown under the Estimates tab. In the top right of the window is a table of calculated statistics for the selected trace.

Tracer will plot a (marginal posterior) distribution for the selected parameter and also give you statistics such as the mean and median. The `95% HPD lower` or `upper` stands for *highest posterior density interval* and represents the most compact interval on the selected parameter that contains 95% of the posterior probability. It can be thought of as a Bayesian analog to a confidence interval.

Select the `treeModel.rootHeight` parameter and the next three (hold shift whilst selecting). This will show a display of the age of the root and the three gene trees. If you switch the tab at the top of the window to **Marginal Density** then you will get a plot of the marginal posterior densities of each of these date estimates overlayed, as shown in Figure 10.

Obtaining an estimate of the phylogenetic tree

BEAST also produces a sample of plausible trees. These can be summarized using the program **TreeAnnotator**. This will take the set of trees and identify

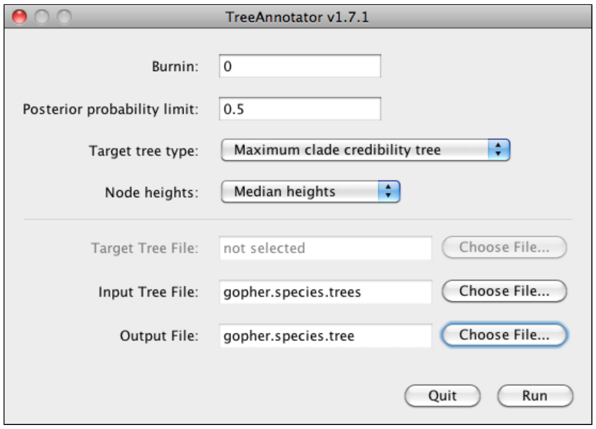

Figure 11: Using TreeAnnotator to summarise the tree set.

a single tree that best represents the posterior distribution. It will then annotate this selected tree topology with the mean ages of all the nodes as well as the 95% HPD interval of divergence times for each clade in the selected tree. It will also calculate the posterior clade probability for each node. Run the **TreeAnnotator** program and set it up to look like in Figure 11.

The burnin is the number of trees to remove from the start of the sample. Unlike **Tracer** which specifies the number of steps as a burnin, in **TreeAnnotator** you need to specify the actual number of trees. For this run, we use the default setting.

The **Posterior probability limit** option specifies a limit such that if a node is found at less than this frequency in the sample of trees (i.e., has a posterior probability less than this limit), it will not be annotated. The default of 0.5 means that only nodes seen in the majority of trees will be annotated. Set this to zero to annotate all nodes.

For **Target tree type** you can either choose a specific tree from a file or ask TreeAnnotator to find a tree in your sample. The default option, **Maximum clade credibility tree**, finds the tree with the highest product of the posterior probability of all its nodes.

Choose **Mean heights** for node heights. This sets the heights (ages) of each node in the tree to the mean height across the entire sample of trees for that clade.

For the input file, select the trees file that BEAST created (by default this will be called `gopher.species.trees`) and select a file for the output (here we called it `gopher.species.tree`).

Now press `Run` and wait for the program to finish.

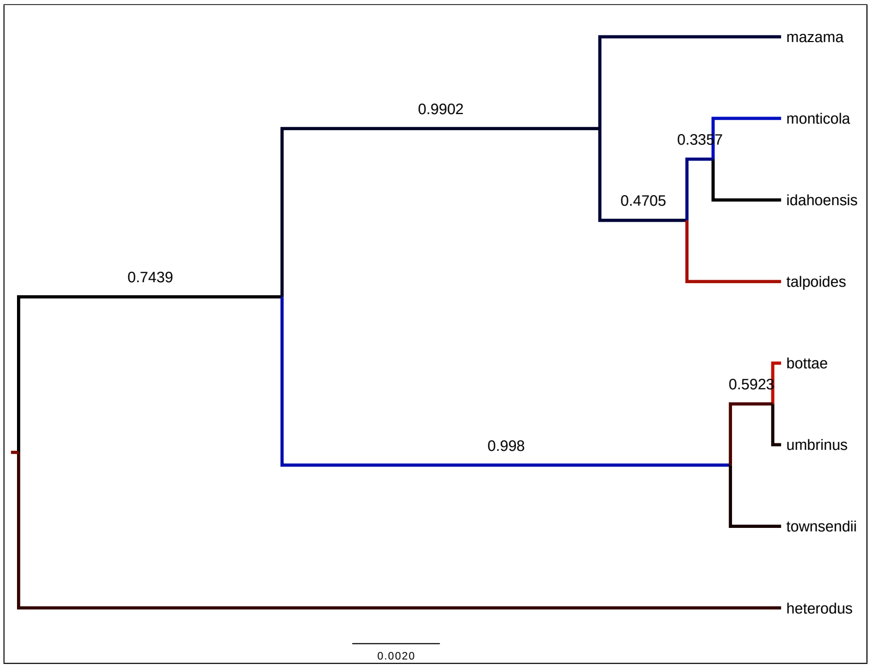

Figure 12: Figtree representation of the species tree.

Viewing the Sprecies Tree

Finally, we can look at the tree in another program called **FigTree**. Run this program, and open the `gopher.species.tree` file by using the Open command in the File menu. The tree should appear. You can now try selecting some of the options in the control panel on the left. Try selecting **Node Bars** to get node age error bars. Also turn on **Branch Labels** and select **posterior** to get it to display the posterior probability for each node. Under **Appearance** you can also tell FigTree to colour the branches by the rate. You should end up with something like Figure 12.

Alternatively, you can load the species tree set into DensiTree and set it up as follows.

- Set burn-in to 500. The tree should not be collapsed any more.
- Show a root-canal tree to guide the eye. Perhaps, the intensity of the trees is not large enough, so you might want to increase the intensity by clicking the icon in the button bar.
- Show clades, their mean and 95% HPD graphically, and posterior support using text. Now, too many clades are shown, and most are not of interest. Select 'Selected only', then open the clade toolbar (menu Window/View

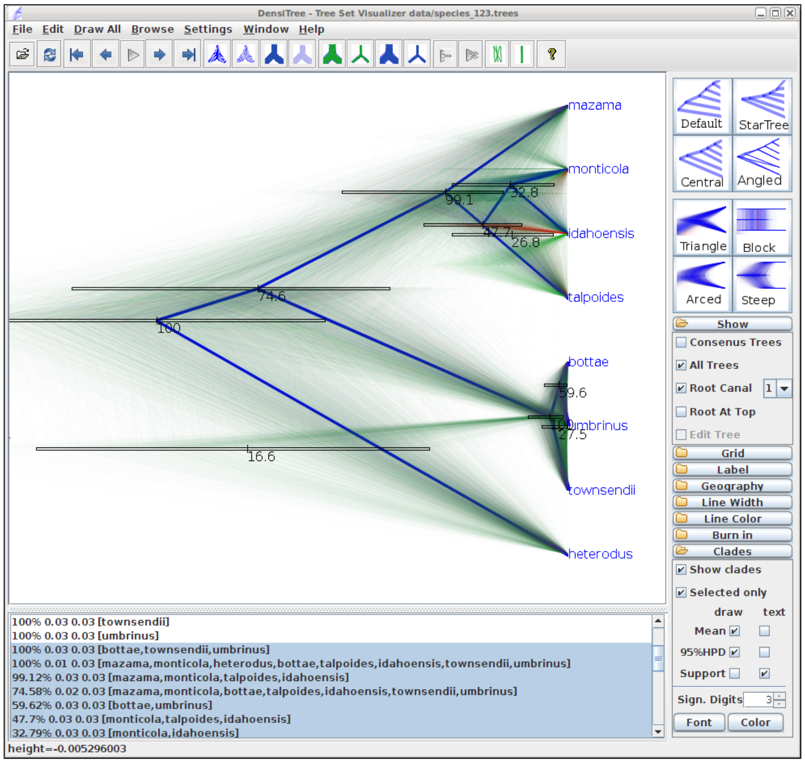

Figure 13: DensiTree representation of the species tree.

clade toolbar), and select only highly supported clades. Also, select the clade consisting of heterodus, bottea, umbinus and townsendii, to show that heterodus is an outgroup, but there is some support (over 16%) that it is not.

- Drag the clade monticola and idahoensis up so that the 95% HPD bar does not overlap with the one for mazama, monticola, idahoensis and talpoidis. Increase font size of the label for better readability.

The image should look something like Figure 13

Exercise: There is about 75% support for heterodus to be an outgroup, and about 17% for heterodus to be in a clade with bottea, umbinus and townsendii. Can you explain where the other 8% went?

DensiTree can be used to show the branch widths of summary tree from tree annotator as population sizes. Under ‘Line Width’ in DensiTree, choose ‘BY_METADATA_NUMBER’ for the bottom and for the top, and choose numbers 2 and 3 in the ’top’ and ’bottom’ spinner. Left, the bottom represents dmv1, the top dmv2 in the summary tree, which do not quite match in areas

with little posterior support for the clades (see Figure 13 to see which clades have little support).

Right, the top is matched up with the bottom of the branch above, using the ‘Make fit to bottom’ option for the top. This looks a bit prettier, but may not be quite accurate.

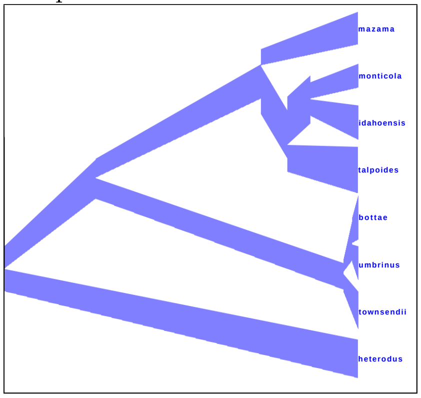

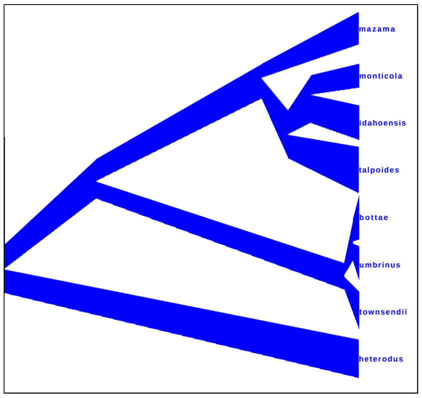

Alternatively, a consensus tree can be generated by biopy (`http://code.google.com/p/biopy/`) with using 1-norm left, and 2-norm right.

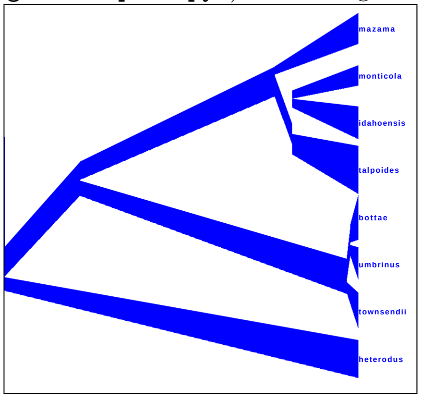

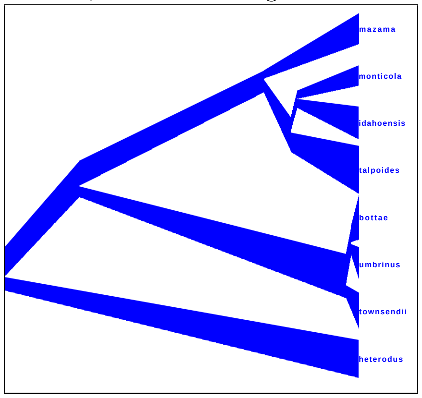

Showing all consensus trees with population widths (use By Metadata Pattern, for top and bottom and use

```
.*dmv=.([^,]*).*
```

for the bottom pattern and

```
.*dmv=.[^,]*,([^\}]*).*
```

for the top pattern. This gives us this visualisation:

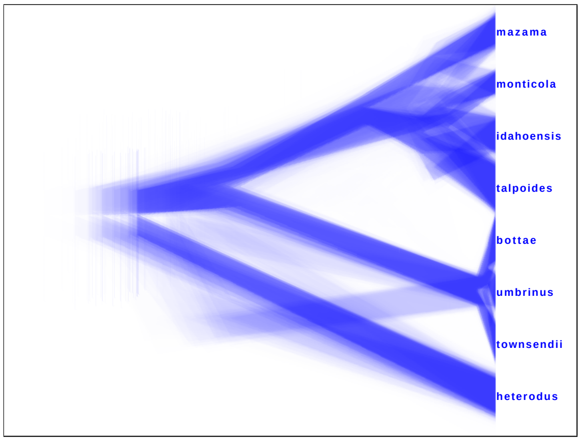

Comparing your results to the prior

Using BEAUti, set up the same analysis but under the MCMC options, select the **Sample from prior only** option. This will allow you to visualize the full prior distribution in the absence of your sequence data. Summarize the trees from the full prior distribution and compare the summary to the posterior summary tree.

References

[1] N.M. Belfiore, L. Liu, and C. Moritz. Multilocus phylogenetics of a rapid radiation in the genus Thomomys (Rodentia: Geomyidae). *Systematic Biology*, 57(2):294, 2008.

[2] Joseph Heled and Alexei J Drummond. Bayesian inference of species trees from multilocus data. *Mol Biol Evol*, 27(3):570–80, Mar 2010.
